# Dispersions of Zirconia Nanoparticles Close to the Phase Boundary of Surfactant-Free Ternary Mixtures

**DOI:** 10.1021/acs.langmuir.0c03401

**Published:** 2021-04-02

**Authors:** Andrea Fiorati, Federico Florit, Andrea Mazzei, Stefano Buzzaccaro, Barbara Rossi, Roberto Piazza, Renato Rota, Luigi De Nardo

**Affiliations:** † Department of Chemistry, Materials and Chemical Engineering “G. Natta”, Politecnico di Milano, Piazza Leonardo da Vinci, 32, 20133 Milano, Italy; ‡ INSTM - Local Unit Politecnico di Milano, Piazza Leonardo da Vinci, 32, 20133 Milano, Italy; § Elettra Sincrotrone Trieste, Strada Statale 14 km 163.5, Area Science Park, 34149 Basovizza, Trieste, Italy; ∥ Department of Physics, University of Trento, Via Sommarive 14, 38123 Povo, Trento, Italy

## Abstract

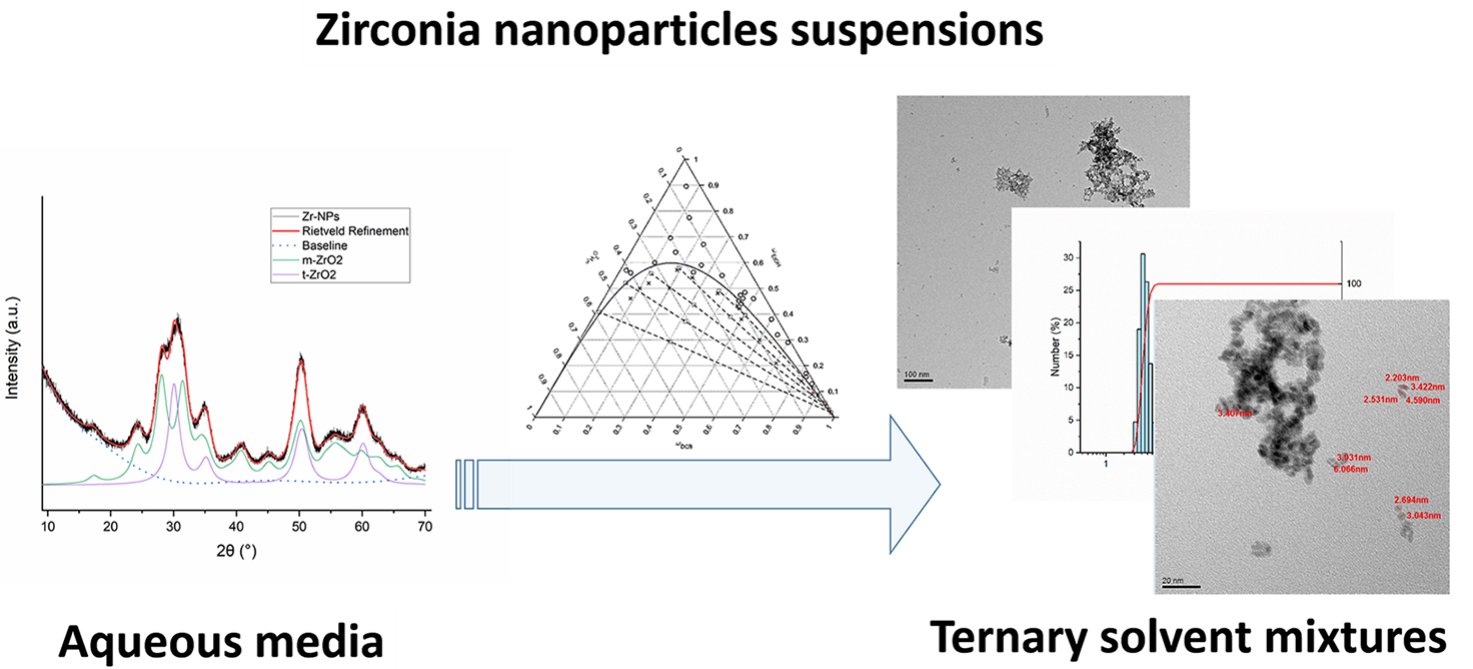

The achievement of a homogeneous dispersion of nanoparticles is of paramount importance in supporting their technological application. In wet processing, stable dispersions were largely obtained via surfactant or surface functionalization: although effective, the use of dispersant can alter, or even impair, the functional properties of the resulting nanostructured systems. Herein, we report a novel integrated modeling and experimental approach to obtain stable ZrO_2_ nanoparticle (NP) dispersions at native dimensions (about 5 nm) in homogeneous ternary mixtures of solvents (i.e., water, ethanol, and 1,2-dichlorobenzene) without any further surface functionalization. A miscibility ternary diagram was computed exploiting the universal quasi-chemical functional-group activity coefficient (UNIFAC) model, which was then experimentally validated. Dynamic light scattering (DLS) on these mixtures highlights that nanometric structures, resembling nanoemulsion droplets, form close to the mixture two-phase boundary, with a size that depends on the ternary mixture composition. ZrO_2_–NPs were then synthesized following a classic sol–gel approach and characterized by XRD and Raman spectroscopy. ZrO_2_–NPs were dispersed in HCl and mixed with different mixtures of ethanol and 1,2-dichlorobenzene (DCB), obtaining homogeneous and stable dispersions. These dispersions were then studied by means of DLS as a function of DCB concentration, observing that the nanoparticles can be dispersed at their native dimensions when the mass fraction of DCB was lower than 60%, whereas the increase of the hydrophobic solvent leads to the NPs’ agglomeration and sedimentation. The proposed approach not only offers specific guidelines for the design of ZrO_2_–NPs dispersions in a ternary solvent mixture but can also be extended to other complex solvent mixtures in order to achieve stable dispersions of nanoparticles with no functionalization.

## Introduction

Zirconium dioxide (ZrO_2_) found widespread application as an engineering ceramic due to its excellent mechanical strength and stiffness, amphoteric behavior, high thermal stability, and dielectric properties.^1−4^ The peculiar properties of ZrO_2_ nanoparticles (ZrO_2_–NPs) have been exploited in a range of applications, encompassing scratch-resistant coatings,^5^ oxygen sensors for fuel cell,^3^ humidity sensors,^6^ and heterogeneous catalysis.^7^ ZrO_2_ can occur in three different polymorphs at atmospheric pressure: the monoclinic phase (m-ZrO_2_), which is the most stable at temperatures below 1400 K, the tetragonal (t-ZrO_2_), which is stable in temperature range 1400–2700 K, and the cubic phase (c-ZrO_2_), more stable at higher temperatures (2700–2950 K).^8^ ZrO_2_–NPs hold the advantage that metastable polymorphs can be dimensionally stabilized at room temperature,^5,9,10^ exploiting the different properties each crystalline phase possess.

The Brownian motion in dispersion of nanoparticles leads to collisions which causes agglomerations^11^ that can be hindered through their electrostatic, steric, or electrosteric stabilization.^12^ The main approach to obtain thermodynamically stable dispersions of ZrO_2_–NPs involves surface functionalization with either surfactants, carboxylic acids with long aliphatic chains, polymers, or other small organic molecules. Due to the amphiphilic behavior of ZrO_2_–NPs, their dispersion in organic solvents generally requires an accurate tuning of the capping agent. Grote and co-worker,^13^ for instance, achieved the stabilization of 10 nm ZrO_2_–NPs in chloroform using hexanoic, decanoic, or dodecanoic acids, with molar ratio (zirconia/additive) up to 10%. Similar results were obtained in tetrahydrofuran by using bifunctional silane coupling agents,^14^ or ligands containing vinyl groups;^15^ vinyl-coated nanoparticles can even be dispersed in acrylate solution and copolymerized with it.^16^ Wang and co-workers, by means of Hansen solubility parameter analysis, investigated the dispersion behavior of carboxylate-grafted ZrO_2_–NPs in 25 different organic solvents, covering a wide range of polarity.^17,18^ Interestingly, they found that the combination of triethanolamine with methacrylic acid broadened the range of compatible solvents from benzene to methanol.^17^

Even if effective in achieving the stabilization of nanoparticles in solvents, the use of additives can alter, by impairing or hindering, the final properties of the systems. Indeed, while surfactants can stabilize colloidal dispersions, they can also add chemical or physical functions to the colloid itself.^19^ In addition, the chemical nature of dispersant strongly influences the surface chemical properties of zirconia nanoparticles.^20^ As an example, the surface treatment of nanoparticles in inorganic–organic composites leads to decreases of the optical properties.^21^ Finally, the addition of different chemical agents alter the viscosity properties.^22^ For these reasons, the development of new strategies to get homogeneous nanoparticle distribution, by avoiding surface capping or functionalization, represents an attractive challenge.^21^

Recently, the intrinsic behavior of the ternary mixture of solvents, composed by two almost immiscible components (water and a hydrophobic organic solvent) and one hydrotope,^23^ has become of great interest for the scientific community.^24^ Indeed, it is reported that when some ternary mixtures are close to the two-phase boundary, the formation of nanometric assemblies can be observed. These systems are commonly defined detergent-less microemulsions, surfactant-free microemulsions, or even ultraflexible microemulsions.^24−27^ In this context, the potential application, e.g., solubilization processes,^27^ of these surfactant-free microemulsions still represent a little explored field.

In this work, we report a novel approach to obtain a thermodynamically stable dispersion of zirconia nanoparticles at native dimensions (about 5 nm) in ternary homogeneous mixtures of three different solvents: water, ethanol, and 1,2-dichlorobenzene (DCB). Since DCB is a hydrophobic solvent, and its miscibility with water is negligible, ethanol, acting as hydrotrope, was added to obtain homogeneous mixtures.^23^ In order to establish the proper ratio between the solvents, a miscibility ternary diagram was computed by means of the universal quasi-chemical functional-group activity coefficient (UNIFAC) model,^28^ a group-contribution thermodynamic model for the estimation of the activity coefficient in mixtures taking into account nonidealities. Contrarily to other activity coefficient models (e.g., NRTL, UNIQUAC), the UNIFAC model only requires the knowledge of the species in the mixture.^29,30^ For this reason, the UNIFAC model is widely applied for miscibility problems when few or no data is available for the analyzed mixture. The computed miscibility diagram was validated empirically and analytically. The ratio between H_2_O, ethanol, and dichlorobenzene, was determined by ^1^H NMR analysis. Once a valid approach to obtain macroscopically homogeneous ternary mixtures was assessed, ZrO_2_–NPs were synthesized by adapting a classical nonaqueous sol–gel approach and characterized them by XRD and Raman spectroscopy. Despite that these nanoparticles can be easily dispersed in aqueous HCl solution (0.1 M), homogeneous dispersions in pure nonpolar solvents like DCB cannot be achieved. This issue was overcome by the employment of the ternary mixtures of solvents studied with the UNIFAC model. ZrO_2_–NPs were first dispersed in HCl 0.1 M and then mixed with a proper amount of ethanol and DCB achieving the desired stable nanoparticle dispersions, and their behavior was then studied by means of dynamic light scattering analysis as a function of 1,2-dichlorobenzene concentration.

## Experimental Section

All chemicals were purchased from Sigma-Aldrich (Sigma-Aldrich, Italy) and used as received without further purification.

## Miscibility Studies on Ternary Mixtures of Water, 1,2-Dichlorobenzene, and Ethanol

### UNIFAC Model

The UNIFAC model considers each molecule as an ensemble of groups, and all groups in each molecule can interact with the ones of other molecules, giving rise to the miscibility properties, expressed by means of activity coefficients. Proper parameters are used to describe the interaction between groups. These are collected in databanks^31^ which are continuously expanded as more experimental evidence is collected. Currently, two sets of parameters are widely used, namely, the standard parameters set,^32^ which is mainly applied for the equilibrium of a liquid and a vapor phase (VLE), and the Magnussen parameters set,^33^ which is applied for the equilibrium of two liquid phases (LLE). In this work, the considered mixtures are high-boiling and can give rise to two liquid phases in equilibrium. The Magnussen set of parameters (UNIFAC-LLE) is therefore used in the following. The UNIFAC-LLE model adopts the standard UNIFAC equations^28^ and solely change the parameters used in the model. Furthermore, the temperature at which equilibrium is considered should range approximately between (10 and 40) °C, as the group-interaction parameters were evaluated mainly in this range.^33^ The aim of the model is to find all the mixtures which lead to phase separation, as to obtain the mixability regions in a ternary diagram. To do so, all regions of the ternary diagram which lead to phase separation are computed via equilibrium calculations adopting the UNIFAC model. Detailed description of the UNIFAC model and the equations needed to compute the activity coefficient are reported in the Supporting Information. In the following, the composition of the mixtures will be expressed using mass fractions:
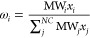
1where MW_*i*_ is the molecular weight of species *i*, *x*_*i*_ the molar fraction of species *i*, and NC the number of species in the system.

### Experimental Validation of the Model

In the first case, the miscibility of the solvents was qualitatively evaluated by adding DCB to homogeneous mixtures of ethanol and water as described in Table S3; then the samples were visually inspected to observe phase separation phenomena and compared with the computed miscibility region. For quantitative validation of the model, 5 different solvent mixtures were prepared: after vigorous mixing, the samples were centrifuged (10 min at 2000 rpm), the two phases were accurately separated, then 80 mg of each phase was diluted in DMSO-d6 (0.750 mL) containing tetramethylsilane (TMS, 0.03%) as an internal standard, and the molar ratios of the solvents were measured by ^1^H NMR spectroscopy by integrating the solvents signals with respect to the internal standard. The molar ratios were successively converted in mass ratios.

^1^H NMR spectra were recorded on Bruker ARX 400 instrument operating at the ^1^H resonance frequency of 400 MHz. Chemical shifts (d, ppm) are reported relative to tetramethylsilane (TMS) as the internal standard. All the spectra were recorded in DMSO-d6 at 305 K. Coupling constants (*J*) are reported in Hz.

As reported in literature,^34^^1^H NMR signals were attributed as follows: H_2_O δ = 3.70 (bs, 2H); EtOH δ = 1.09 (CCH_3_, t, 3H), 3.45–3.54 (CCH_2_O, dq, 2H, *J* = 5.09, 6.99, and 14.06 Hz), 4.38 (OH, t, *J* = 5.09); DCB δ = 7.35–7.42 (CHCHCH, m, 2H), 7.60–7.66 (ClCCHC, m, 2H). Due to the high concentration of the samples, as a consequence, chemical shifts of EtOH and H_2_O signals may show some drift.

## Preparation of Zirconia Nanoparticles Dispersion in Ternary Mixture

### Synthesis of Zirconia Nanoparticles

The synthesis of nanoparticles was carried out by adapting the nonaqueous sol–gel approach from literature,^35,36^ and all of the reactions were conducted in a sealed pyrex tube under air atmosphere. Zirconium(IV) *n*-propoxide solution (70% in *n*-propanol, 3.5 mmol, 1.6 mL) was added to benzyl alcohol (BnOH, 10 mL) in a 50 mL pyrex tube under magnetic stirring. After sealing, the reactive mixture was heated at 200 °C and left to react for 6 days. At the end of the reaction, the reactive mixture was cooled down to room temperature and the resulting suspension was centrifuged for 45 min at 4000 rpm. The collected white powder was washed twice by suspending it in absolute ethanol (20 mL) and centrifuging it for 45 min at 4000 rpm. After the washing, 280 mg (2.3 mmol, *y* = 65%) of zirconium oxide nanoparticles (ZrO_2_–NPs) were obtained.

### Dispersion in Ternary Mixtures Preparation and Characterization

ZrO_2_–NPs in a ternary mixture was prepared as follows. The proper amount of ethanol wet ZrO_2_–NPs were dispersed in aqueous HCl (0.1 M) and then mixed for a few minutes by magnetic stirring until a homogeneous and clear stock dispersion was achieved, containing 9.4 g L^–1^ of nanoparticles. Then a small volume of this dispersion was diluted with other aqueous HCl (0.1 M), ethanol, and 1,2-dichlorobenzene to achieve the mass ratios summarized in Table 1. For the preparation of 10 mL of TM1, e.g., 119 μL of ZrO_2_–NPs stock dispersion, was diluted with 1.79 mL of HCl_aq_ (0.1 M), in a vial under stirring, then 7.10 mL (5.60 g) of EtOH was added, and finally, 1.02 mL (1.33 g) of DCB was dropped into the solution.

**Table 1:** Mass Fraction, Density, and Viscosity of the Ternary Mixtures of Solvents Used for ZrO_2_–NPs Dispersions

mixture ID	ω_DCB_ (%)	ω_EtOH_ (%)	ω_HCl_ (%)	ρ (g mL^–1^)	η (mPa s^–1^)	*n*
TM1	15.07	63.61	21.32	0.889 ± 0.01	1.78 ± 0.06	1.3818
TM2	60.23	37.74	2.03	1.04 ± 0.02	1.24 ± 0.04	1.4542
TM3	82.15	16.84	1.01	1.17 ± 0.02	1.22 ± 0.04	1.5018

### Characterization

Zirconia nanoparticles were characterized by X-ray diffraction experiments (XRD), conducted with a Panalytical Empyrean diffractometer using the Bragg–Brentano geometry (Cu Kα1 radiation; λ = 0.154056 nm). The X-ray diffraction patterns were collected at room temperature in 5–70° 2θ range (scan step size = 0.02°, scanning time as per step = 20 s). The measure was repeated 3 times in order to increase the signal-to-noise ratio.

Raman spectra were recorded on ZrO_2_ NPs placed on a glass slide, in air, at room temperature using an integrated micro-Raman system (Horiba–Jobin–Yvon, LabRam Aramis). The exciting radiation at 632.8 nm provided by the emission of a He–Ne laser was focused onto the sample surface with a spot size of about 1 μm^2^ through a 100× objective. The scattered radiation was analyzed using a 46 cm focal length spectrograph equipped with a holographic grating with 1800 grooves mm^–1^ and a charge-coupled device (CCD) detector. The Rayleigh scattering was filtered through a narrow band edge filter. The resolution was set to about 0.35 cm^–1^/pixel. The Raman spectra were recorded on the same sample several times to ensure the reproducibility of the measurements and to exclude any possible photodegradation effect.

The densities (ρ) at 25 °C of TM1, TM2, and TM3 were evaluated by weighing 5 mL of the mixtures previously measured in a calibrated flask; the viscosities (η) at 25 °C were measured by means of a modified Ubbelohde viscometer (all these measurements were repeated 5-fold).

Dimensions of ZrO_2_–NPs were determined by dynamic light scattering (DLS) measurements, conducted on a Zetasizer Nano ZS instrument (Malvern, UK), at 25 °C and 632.8 nm, with an equilibration time of 120 s at a scattering angle of 173°. After the synthesis, the ZrO_2_–NPs were dispersed in aqueous HCl (0.1 M) achieving clear dispersions, which were then transferred to Suprasil quartz glass cuvette and directly analyzed. The DLS measurements of the ternary mixtures were conducted on a custom-made dynamic light scattering setup (wavelength = 532 nm, scattering angle = 90°) that allows a better characterization of the short-time dynamics of the sample. In addition, this setup is equipped with a special cell that allows an optimal filtration of the sample, which strongly reduces the presence of spurious artifacts in the intensity correlation function due to the presence of dust.

The concentration of ZrO_2_ in the solutions were determined by inductively coupled plasma-optical emission spectrometry (ICP-OES, PerkinElmer Optica 8300), and the samples were analyzed after a microwave-assisted digestion with nitric acid (65% in water, trace metal grade).

Ten microliters of the dispersion were deposited on a 200-mesh carbon-coated copper grid and dried under ambient condition before analysis. ZrO_2_–NPs were analyzed by transmission electron microscopy (TEM, Philips CM 200 field emission gun). High-resolution TEM (HR-TEM) was performed by using a 200 kV accelerating voltage. Low beam current densities and short acquisition times were adopted in order to avoid structural transformation during acquisition of HR-TEM images.

## Results and Discussion

### Miscibility Studies on Ternary Mixtures of Water, 1,2-Dichlorobenzene, and Ethanol

Due to its intrinsic chemical-physical properties, water is generally poorly soluble, or in some case virtually insoluble, in nonpolar organic solvents like 1,2-dichlorobenzene (DCB). Indeed, when water and DCB are mixed together, they undergo phase separation.^37^ This issue can be easily overcome by the addition of a proper amount of a third polar cosolvent, which is totally miscible with both the species. In this work, ethanol (EtOH) was chosen as a cosolvent to achieve homogeneous mixtures with high mass fraction of DCB suitable for obtaining ZrO_2_–NP dispersions at their native size. While using high mass fraction of EtOH (*e.g*., *ω*_EtOH_ ≳ 0.6), homogeneous solutions of the selected three solvents can be easily achieved, a reduction of the ethanol mass fraction below *ω*_EtOH_ ≲ 0.6 generally leading to phase separation.

**Figure 1:**
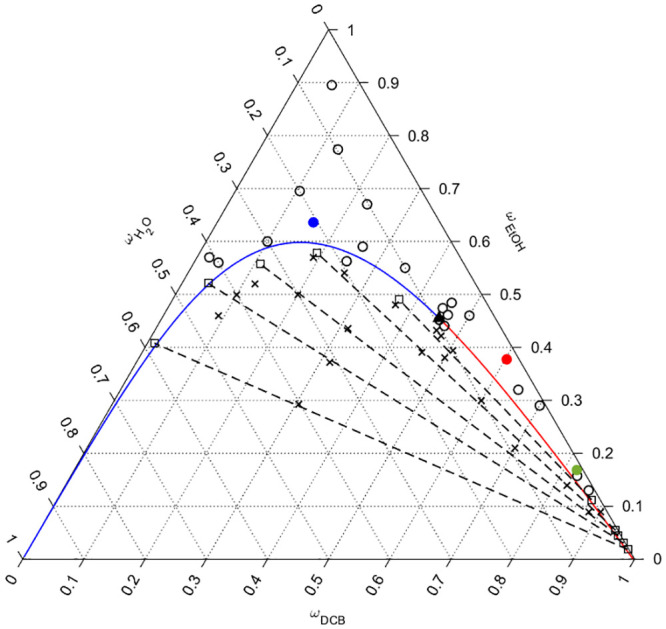
Ternary miscibility diagram of H_2_O, ethanol, and 1,2-dichlorobenzene mixtures as computed from the UNIFAC-LLE model. Continuous blue and red lines represent the boundaries of the mixability region (phase separation below these lines). Circles and × represent the qualitative empirical validation test. Circles indicate the mixtures which do not experimentally lead to phase separation, while × indicate mixtures which show phase separation. Squares indicate the mixtures prepared to determine the tie lines (dashed lines) as from ^1^H NMR. The three colored circles (blue, red, and green) correspond to the composition of the three solvent mixtures discussed in Table 1, respectively TM1, TM2, and TM3. The black triangle is the predicted critical point of the mixability region (color figure online).

To accurately predict the proper mass fraction of each solvent, the UNIFAC model was applied and its reliability was experimentally tested with two different approaches. The UNIFAC model was chosen because the solvent mixture is fully defined, but no phase-separation data was available for this specific mixture. Notably, the UNIFAC model only requires the knowledge of the structure of the chemicals involved, while other models (NRTL, UNIQUAC) would require an extensive experimental campaign aimed at finding the required model parameters.^29,30^ Instead, the UNIFAC model does not require user-provided parameters. In order to apply this thermodynamic model, the molecules involved in the mixture should be described by groups of atoms which establish specific interactions between them as described by the model through proper group-interaction parameters. The set of equations defined in UNIFAC model section and in the Supporting Information was numerically solved using the parameters reported in Table S1, and the phase diagrams reported in Figure 1 and Figure S13 were obtained.

The model correctly predicts the miscibility of ethanol and DCB and the insolubility of water in DCB. The zone below the continuous line in the ternary diagram is in fact a miscibility gap (Figure 1): all mixtures having a composition within this zone will always lead to phase separation. The model can predict the composition of the two phases which form after separation, and they are represented in the diagram as dotted lines (tie lines). All mixtures, which composition lies on a tie line, will lead to the same composition of the two separated liquid phases.

The qualitative model validation was carried out as described in experimental validation of the model section. Figure 1 additionally reports the results of the validation procedure. The circles represent the ternary mixtures which results in homogeneous solutions, while crosses represent the ternary mixtures which undergo phase separation: in all cases, the applied UNIFAC model well describes the ternary solvent mixture in terms of phase separation. As a further quantitative confirmation, 5 different solvent mixtures were prepared to obtain phase separation (Table S4) and the phase mass fractions were measured by means of ^1^H NMR analysis (Figures S1–S12). The results of ^1^H NMR titration, reported in Table S4, and in Figure 1 as squares, show a good accordance with the predicted tie lines. The position of the aqueous (low DCB fraction) phases on the diagram is more correctly predicted than the organic (high DCB content) phases. The different isomers of DCB are significantly different between each other in their physical properties, e.g., 1,4-dichlorobenzene is solid at ambient temperature. This means that complex interactions arise between DCB molecules, according to the position of the chlorine atoms on the aromatic ring. The used UNIFAC model does not take into account the position of the chlorine atoms in the molecule, and currently, no group contribution is available in the literature for groups containing chlorine and aromatic carbons separated by 0, 1, or 2 further aromatic carbons.^31^ The accuracy for the aqueous phase can be explained in the same manner; as the quantity of DCB is very low, the overall influence of the inaccuracy in describing DCB isomers becomes negligible. The aqueous phase is thus described as a mixture of water and ethanol with traces of an organic aromatic species (regardless of its actual structure).

Overall, the UNIFAC model provided a satisfactory prediction of the miscibility properties of the selected mixture of solvents. Notably, this model can be used for any combination of solvents (not limited to relatively simple compounds as the ones used in this work) and for any number of components. The UNIFAC model can be adopted also for more complex ternary mixtures, such as those for which more than one binary mixture shows a miscibility gap.^33^ This model is thus a useful tool to screen possible solvent mixtures (which could lead to phase-separation) for the subsequent dispersion of nanoparticles, without the need to prepare a high number of solutions aimed only at finding the miscibility properties of the proposed solvents.

As the nanoparticles are dispersed in HCl_aq_ (0.1 M), the same qualitative tests were run by using a 0.1 M aqueous solution of HCl instead of pure water. No macroscopic difference was observed in the miscibility behavior of the mixtures. Therefore, the addition of a small quantity of HCl does not produce significant differences with respect to the case of pure water in terms of miscibility of the three solvents. This result is consistent with the results presented by Lopian and coauthors, where they investigate the effect of strong acid in ternary mixtures made of octanol/ethanol/water.^26^

### Characterization and Dispersion in Acidic Aqueous Solution of ZrO_2_ Nanoparticles

The nonaqueous sol–gel synthesis of zirconium(IV) *n*-propoxide with BnOH results in ZrO_2_ nanoparticles (NPs) with uniform size and both tetragonal (t-ZrO_2_) and monoclinic (m-ZrO_2_) phases:^38^ the t/m-ZrO_2_ ratio can be tuned by varying the reaction temperature, the constituent material of reactors (glass or Teflon), and the scale.^9^ Indeed, the increase of the temperature up to 270 °C leads to a higher amount of tetragonal phase in glass vessels, while the use of the Teflon reactor allows one to achieve similar results at lower temperatures. The results here reported refer to the synthesis performed in a sealed Pyrex glass tube at 200 °C, the main scope of this work being the achievement of homogeneous dispersions.

**Figure 2:**
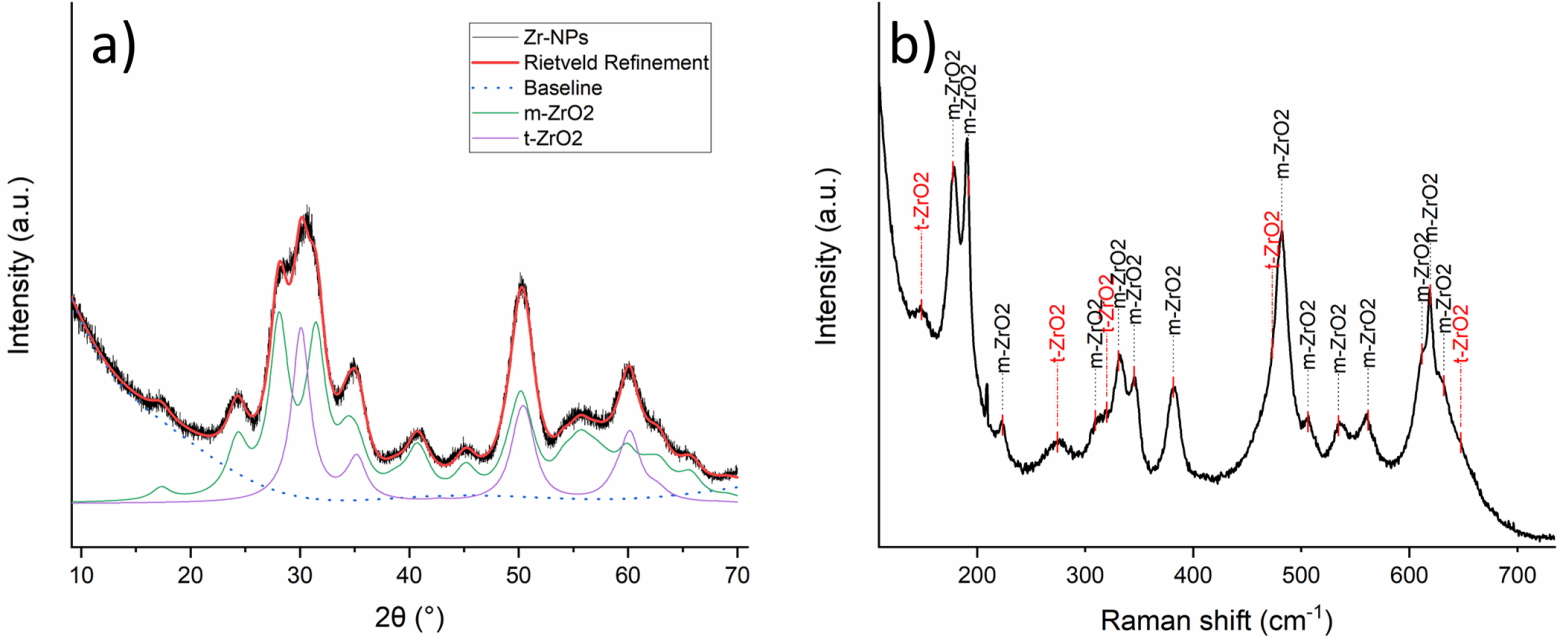
(a) XRD diffraction pattern and Rietveld refinement of ZrO_2_–NPs, where ZrO_2_–NPs diffractogram (Black line), computed Rietveld Refinement (Red line), subtracted baseline (dashed blue line), m-ZrO_2_ (green line computed), and t-ZrO_2_ (purple line computed) XRD patterns. (b) Raman spectrum of ZrO_2_–NPs collected using 633 nm of excitation wavelength.

The crystal phase composition and the crystallite size of the obtained ZrO_2_–NPs were quantified by performing Rietveld refinement (RR) on XRD diffractogram (Figure 1a). RR was performed by means of Profex software^39^ for recalculating the ICSD reference patterns of m-ZrO_2_ (ICSD code: 98–008–0045) and t-ZrO_2_ (ICSD code: 98–006–6789). The recalculated diffractogram (Figure 2a), χ^2^, and GOF (Table S5), indicate the quality of the fitting, the obtained values reliably providing the crystallite size, and the phase composition of the ZrO_2_–NPs. The calculated weight fractions and the crystallite dimensions are reported in Table S5: results are consistent with previous published results; indeed Cheema and co-worker achieved, with the same synthetic approach at slightly higher temperatures, nanoparticles with 80% of m-ZrO_2_ fraction and a crystallite dimension of about 5 nm.^9^ The dominance of the m-ZrO_2_ phase was also confirmed by Raman spectroscopy, this technique being successfully employed to distinguish the ZrO_2_ phases,^40−42^ thanks to its sensitivity to the molecular environment. Indeed, as shown in Figure 2b, in the Raman spectrum of collected nanoparticles, the signals attributed to m-ZrO_2_ were predominant (177, 190, 223, 309, 331, 344, 381, 481, 503, 536, 560, 615, 619, and 631 cm^–1^), while only the peaks at 145 and 277 cm^–1^ can be clearly attributed to t-ZrO_2_ because all the others t-ZrO_2_ signals (319, 472, and 646 cm^–1^) appear only as shoulder of m-ZrO_2_ peaks.^40−42^

**Figure 3:**
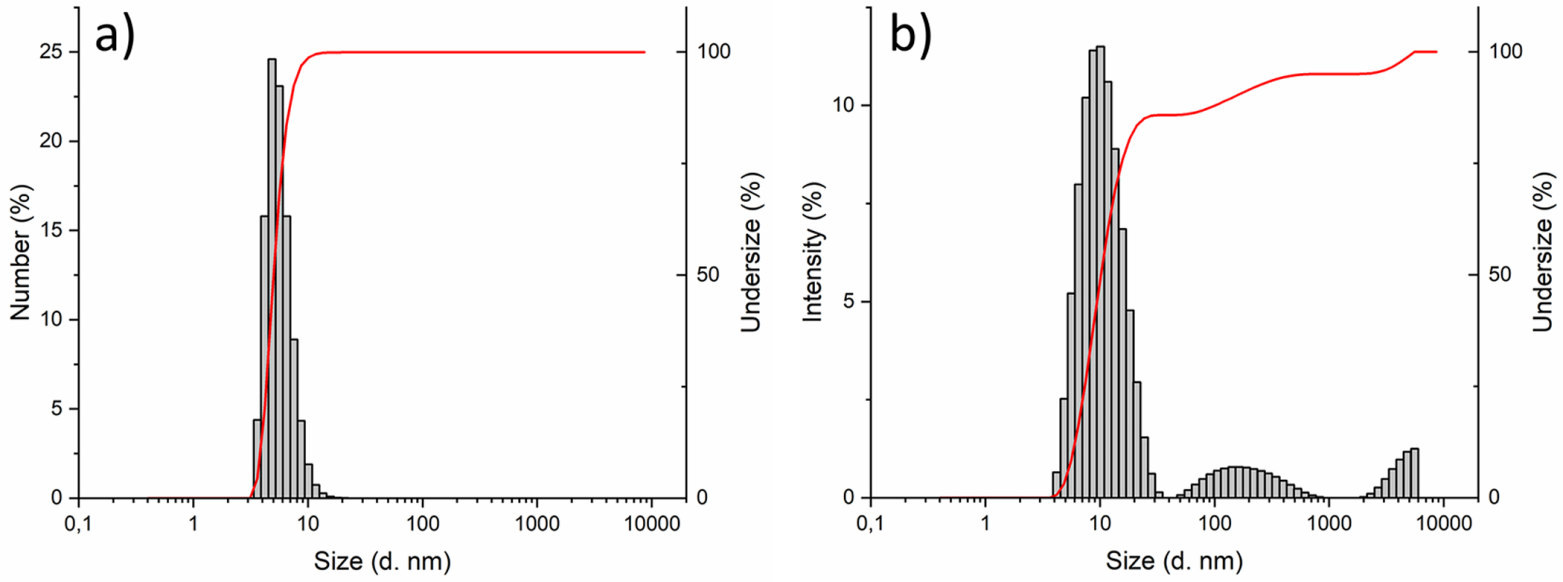
Particles size distribution of ZrO_2_–NPs dispersed in aqueous 0.1 M HCl. (a) Data expressed in number of NPs vs size, (b) data expressed in intensity of scattered light vs size. Gray bars represent the population frequency and red line the cumulative size distribution.

ZrO_2_–NPs have been easily dispersed in aqueous HCl 0.1 M simply by adding the powder to the acidic aqueous solution, resulting in a clear and transparent dispersion with concentration up to 9.4 g L^–1^, without adding any dispersant. After appropriate dilution in aqueous HCl 0.1 M, DLS measurements (number distribution) showed a homogeneous particle size of this dispersion, resulting in a hydrodynamic diameter of about 5 nm (DLS, Figure 3a). By analyzing DLS measurements in terms of intensity distribution (Figure 3b), it is possible to observe additional peaks, emphasizing the presence of larger aggregates with dimensions of hundreds of nanometers. DLS data is in fair accordance with crystallite size measured via XRD and Rietveld refinement (Table S4). Interestingly, this dispersion in water has been obtained without ZrO_2_–NPs functionalization.

In order to widen the range of applications, ZrO_2_–NPs generally need to be dispersed in organic solvents prior to their use: for this reason, we investigated the possibility of preparing ZrO_2_–NP dispersions in homogeneous ternary mixtures composed by water, 1,2-dichlorobenzene, and ethanol, avoiding any chemical modification of the nanoparticles surface. The proposed approach can be in principle extended to other organic solvents.

### DLS Tests of Nanoparticle Dispersibility in Ternary Mixtures

With the aim of exploiting whether the zirconia nanoparticles can be dispersed into a nonaqueous solvent, we choose to approach the lower-right corner of the phase diagram in Figure 1 by following a path that borders the mixture two-phase boundary. Specifically, we made a detailed DLS test of the dispersibility and stability of the ZrO_2_ nanoparticles at a concentration of about 0.11 g L^–1^ in the three solvents indicated by the blue, green, and red dots in Figure 3, whose compositions can be found in Table 1.

Before discussing the DLS result, it is useful to point out that a comparison of the visual appearance and time evolution of the three zirconia dispersions already highlights noticeable differences. Indeed, while TM1 and TM2 are transparent, without any evident flocculation up to several weeks since preparation, sample TM3 rapidly shows the formation of a sediment at the bottom of the cuvette. Visual evidence seems therefore to suggest that a consistent fraction of NPs may remain dispersed even upon a reduction of the water content to about 2% in weight (solvent TM2). On the other hand, the rapid settling observed in sample TM3, whose water content is not much lower, seems to imply that, to keep stable the dispersion, the presence in the solvent of a substantial fraction of a polar component (like ethanol) is needed.

**Figure 4:**
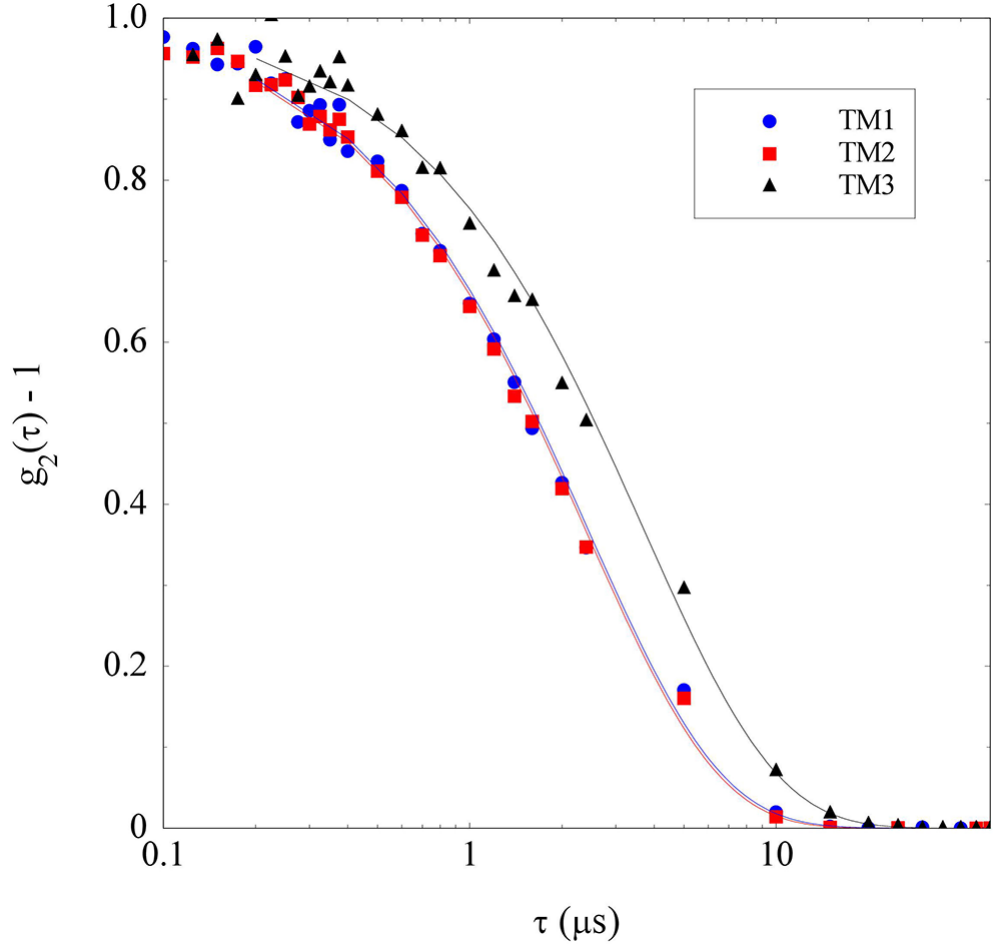
Intensity correlation functions *g*_2_ (τ) from the ternary solvents mixtures indicated in Table 1 fitted with single exponentials.

A more quantitative assessment of the previous considerations can be obtained by DLS. Yet, the analysis of scattering data from the investigated dispersions is not trivial because, rather surprisingly, the selected solvents significantly contribute both to the scattered intensity and to the decay of the DLS correlation functions. A straightforward reconstruction of the particle size distribution similar to the one shown in Figure 2 would in fact suggest the presence of scatterers that are consistently smaller than the original NPs. Hence, we regarded as useful to perform a DLS investigation of the ternary solvent mixtures used for the dispersions (TM1, TM2, and TM3). As shown in Figure 4, the correlation functions for the solvent mixtures decay on a time scale of a few microseconds, which is far larger than those typical of simple liquid mixtures. Notably, *g*_2_ (τ) is similar for the three solvent compositions investigated and can reasonably be fitted as a single exponential, *g*_2_ (τ) – 1 = exp(−τ/*τ*_*r*_), with the same characteristic time *τr* ≃ 2.4 μs for both TM1 and TM2 and *τr* ≃ 3.5 μs for TM3 (Figure 4).

Considering the values for the solvent viscosities given in Table 1, these relaxation times yield a characteristic size (radius) for the scattering structures observed in solvents TM1, TM2, and TM3 of about 0.3, 0.5, and 0.8 nm, respectively. One may guess that these values correspond to the correlation length ξ of the solvent, whose value could be enhanced by the presence of critical fluctuations. Within this interpretation, however, it is rather hard justifying that the largest value of ξ is obtained for the sample that is farther from the critical point (see Figure 1). Besides, appealing to a consistent contribution of the critical fluctuations implies assuming that the coexistence curve is very close to the spinodal line bordering the region of thermodynamic instability of the mixture.

A possible alternative explanation is that the observed correlations are due to the so-called “pre-Ouzo effect”, a self-aggregation effect that has been reported for a wide class of ternary mixtures composed by a “hydrotrope” (such as ethanol) and two mutually immiscible fluids (like water and DCB), both soluble in the hydrotrope in any proportions.^23,43,44^ This peculiar phenomenon is named after the better known and widely investigated Ouzo effect, which amounts to the formation of rather monodisperse surfactant-free emulsions upon phase separation in ternary mixtures of the same kind. The crucial difference is that the pre-Ouzo effect, whose origin is still debated,^45,46^ does not occur within the phase coexistence but rather within the stable region of the phase diagram.

Distinguishing between these two different interpretations will necessarily require a more extensive investigation of the solvents we used, arguably by means of techniques allowing to explore a much wider range of scattering wave-vectors such as small-angle X or neutron scattering. Nevertheless, as discussed in the following, this anomalous scattering effect must be attentively considered in the analysis of the DLS correlation functions from the particle suspensions.

We now consider DLS measurements of the samples prepared in solvents TM1 and TM2 that, as discussed above, visual inspection suggests to be rather stable dispersions. The “bare” correlation functions originally obtained from the samples were first cleared of the solvent contribution by focusing on the short-time behavior of the field correlation function . The latter was regarded as a linear combination of the decay due to the NPs plus a faster contribution due to the “nanodroplets” spontaneously occurring in the solvent using the droplet size obtained from the data in Figure 4. This numerical procedure allowed us to estimate a solvent contribution to *g*_1_ (τ) amounting from 20% for the TM2 dispersion up to to 48% for the TM1 sample, which can then be accurately subtracted out with the effect of modifying the decay rate of the correlation function on time scales shorter than a few tens of microseconds.

By taking into account the effect of the solvent viscosity on the decay of *g*_2_ (τ) and of its refractive index on the scattering wave-vector, these “corrected” intensity correlation functions can be directly compared with the correlation function obtained for the original aqueous NPs dispersion.

**Figure 5:**
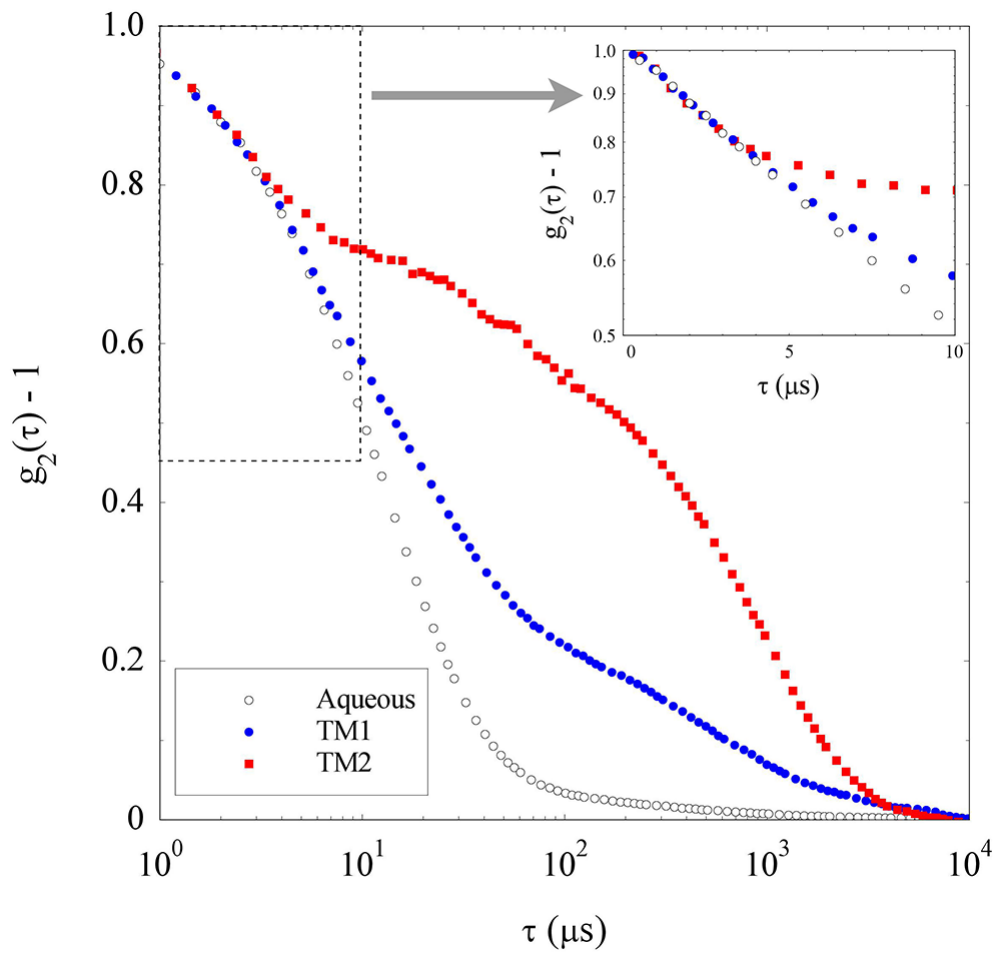
Intensity correlation functions of the zirconia dispersions in solvents TM1 (blue ●) and TM2 (red ■) obtained by subtracting the solvent contribution with their time axis rescaled as described in the text, compared to the correlation function for the nanoparticles in the original aqueous solvent (H_2_O + 0.1 M HCl, ○). The short-time region bounded by the dotted rectangle is expanded in the inset on a log *y*-scale.

Figure 5 shows that the three displayed correlation functions share a common general shape, characterized by a fast initial decrease followed by a much slower decay, whose fractional amplitude is very limited for the aqueous sample but becomes consistently more relevant for the TM1 sample and becomes the dominant contribution for the dispersion in the TM2 mixture. This slow-decay component can be easily attributed to the presence of NPs’ aggregates that were already detected for the original aqueous dispersion (see Figure 3), but that progressively get larger and arguably more numerous by exchanging the solvent to TM1 and, even more, TM2. The Figure 5 inset nevertheless shows that the short-time decay is basically identical for the three correlation functions, witnessing the persistence on nonaggregated NPs both in TM1 and TM2.

Given that the aggregate contribution is by far the dominant contribution to the decay of *g*_2_ (τ), one might however guess that the residual fraction of NPs, dispersed at their native dimensions, in sample TM2 is negligible. Yet, this first impression is fallacious, being essentially due to the strong dependence of the scattered intensity on the particle size. Indeed, provided that the NP clusters can be regarded as rather compact (namely, not tenuous fractal) objects of size *R*_*c*_, their fractional contribution to the scattered intensity scales as *c*_*c*_*R*_*c*_^3^, where *c*_*c*_ is the fraction of NPs aggregated into the clusters. As detailed in the Supporting Information, the typical cluster size and a rough evaluation of *c*_*c*_ can be obtained by considering the average relaxation time of *g*_2_ (τ), defined as the time-integral of the correlation function and by subtracting out the particle contribution (details are reported in the Supporting Information, section S4). The result of this approximate numerical analysis shows that the cluster size progressively increases from *R_c_* ≃ 120 nm in water to *R*_*c*_ ≳ 300 nm in both TM1 and TM2. However, even considering the approximation made in the evaluation, in both cases the fraction of particles associated in clusters is smaller than one part over ten thousand (Table S5).

**Figure 6:**
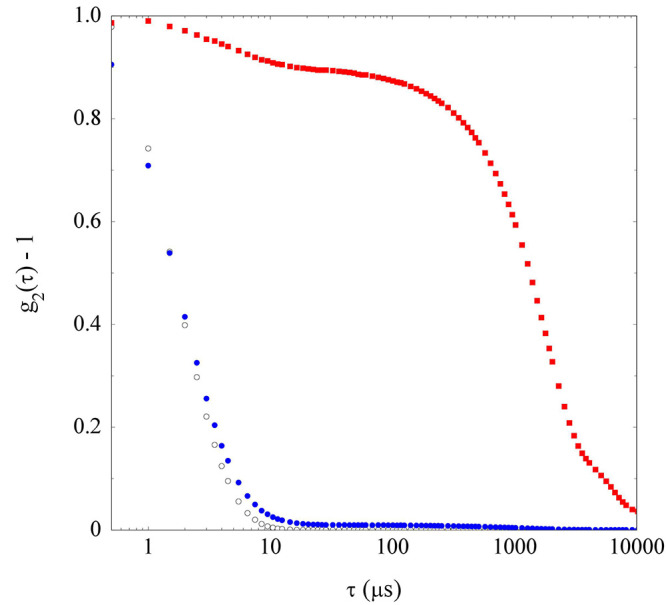
Intensity correlation functions of the zirconia dispersion in solvent TM3 just after preparation (red ■) and after filtration with a PTFE 0.1 μm filter (full ●), compared to the correlation function for the solvent mixture TM3 (○).

Measurements from the macroscopically unstable TM3 dispersion display a totally different scenario. Indeed, when a freshly prepared TM3 dispersion is fed into the light scattering cuvette without filtering, the DLS correlation function, shown in Figure 6, displays the presence of huge and rapidly settling aggregates, which are however almost completely removed by filtering, leaving an almost undetectable amount of NPs in solution. We can then conclude that in solvent TM3 the zirconia NPs undergo a rapid and complete colloidal aggregation process.

**Figure 7:**
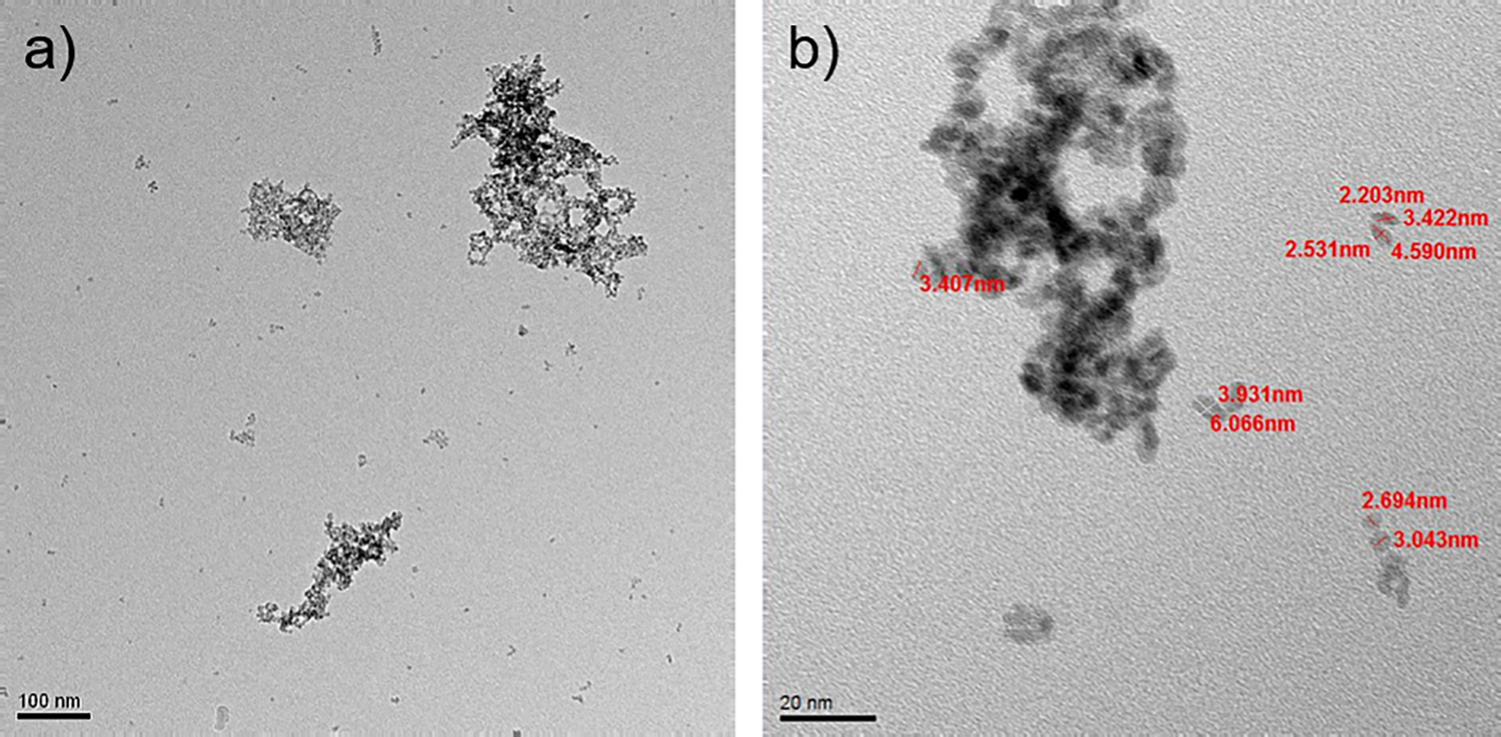
TEM images of sample TM2. Scale bar of (a) and (b), respectively, 100 and 20 nm. A HR-TEM image is reported in Figure S14.

Summing up, DLS measurements show that the zirconia nanoparticles keep dispersed in their native size, with a tiny fraction of small aggregates up to a DCB weight fraction of about 60% (solvent TM2) at least. This evidence is confirmed by TEM images (Figure 7), which display the presence of a large number of single ZrO_2_ nanoparticles coexisting with small clusters composed by few particles, whereas bigger aggregates are rarely observed. Further increase of DCB to ω = 82% leads, however, to the rapid growth of large aggregates incorporating almost all the individual nanoparticles.

While the achievement of stable dispersions of ZrO_2_–NPs in organic solvents is rather easily achieved by the superficial modification of the nanoparticles, obtaining dispersions where the particles retain their native size is anything but trivial. At the same time, the stability of the dispersions is of great importance from an applicative point of view. In the case of ZrO_2_–NPs dispersed after surface functionalization, the stability of the dispersion can strictly depend on the kind and degree of grafting and on the storing conditions. For instance, the stability of dispersions made of ZrO_2_–NPs functionalized with vinyl group-containing ligands can encounter flocculation when the grafting degree is below a certain value or when stored in an open vessel.^15^

### Outlook

Despite herein we are offering specific guidelines for the design of zirconia-based nanodispersion in 1,2-dichlorobenzene, the employed approach can be further extended to other systems. Indeed, thanks to the availability of the group-contribution parameters of many functional moieties, the UNIFAC-LLE model equations can be numerically solved for a high number of common solvent mixtures. Consequently, once a solvent able to disperse nanoparticles at the desired concentration is identified, this dispersion can be diluted with other solvents, which are theoretically unsuitable to achieve homogeneous dispersions, exploiting the information coming from the mixability diagrams computed with the UNIFAC-LLE model. As a first approximation, the model results can be validated qualitatively case-by-case by preparing different solvent mixtures and visually inspecting it. However, the behavior of final dispersions in terms of aggregates dimensions cannot be easily predicted *a priori*. For example, in the case of ZrO_2_-NPs dispersed in aqueous HCl, the substitution of EtOH with acetone leads in all cases to nanoparticle aggregation and consequent precipitation (data not shown).

## Conclusions

Zirconia nanoparticles were synthesized following a classic sol–gel approach widely reported in the literature, and the achieved nanoparticles were characterized by means of X-ray diffraction and Raman spectroscopy. The synthesized ZrO_2_–NPs resulted in having crystallite dimensions of about 4 nm and were a mixture of monoclinic and tetragonal phases (respectively, 69% and 31%). These nanoparticles could be easily dispersed in aqueous HCl (0.1M) at their native dimensions; indeed, DLS measurements showed particles with a hydrodynamic diameter of about 5 nm.

Several applications of ceramic nanoparticles require their effective dispersion in an organic solvent, often immiscible with water. In this work, we overcame the use of additives employing a mixture of three different solvents. 1,2-dichlorobenzene is a chlorinated solvent which is generally immiscible with water, but by means of a polar cosolvent, like ethanol, it was possible to generate homogeneous ternary mixtures with water, when all the solvents are mixed in the proper amount. In order to predict which solvent ratios were able to form stable and homogeneous solutions, a ternary miscibility diagram of H_2_O, ethanol, and 1,2-dichlorobenzene mixtures was computed from the UNIFAC-LLE model and was experimentally validated. DLS analysis highlighted that, when the mixtures are closed to the two-phase boundary, nanometric structures were formed, as in the case of surfactant-free microemulsions, and as far as we know, this result is reported for the first time for the three chosen solvents. The good accordance between the results computed from UNIFAC-LLE and the experimental results, also in the presence of surfactant-free microemulsions, confirm once again the flexibility of this model. Then, the behavior of the ZrO_2_–NPs dispersed in the ternary mixtures were studied by means of DLS experiments, which indicated that the obtainment of nanoparticles dispersed at their native dimensions is possible when the DCB mass fraction was lower than 60%. However, increasing the amount of 1,2-dichlorobenzene generally leads to rapid and complete nanoparticles aggregation, in particular, when the mass fraction reaches values of about 80%.

Thanks to the flexibility of the UNIFAC-LLE model, this approach can be easily extended to other ternary mixtures of solvents, in order to tune the solvent mixture according to the specific needs of the desired applications and can be used for nanoparticle dispersions by avoiding the addition of any other dispersant or further surface modifications.

## Supporting Information Available

Details on UNIFAC model equations and UNIFAC-LLE parameters, details on mixtures prepared for the validation of the UNIFAC model, ^1^H NMR analysis, ternary miscibility diagram and solvent mixtures, crystalline properties, HR-TEM, field correlation functions, and DLS analysis (PDF)
