# Supplementary material for: Dispersions of Zirconia Nanoparticles Close to the Phase Boundary of Surfactant-Free Ternary Mixtures
Source: Langmuir. 2021 Apr 2;37(14):4072–81. doi: 10.1021/acs.langmuir.0c03401 (PMC8154878; doi:10.1021/acs.langmuir.0c03401)
Supplement: Supplementary file 1 — la0c03401_si_001.pdf [file la0c03401_si_001.pdf]

Dispersions of zirconia nanoparticles close to the phase boundary of surfactant-free ternary mixtures

Andrea Fiorati,[a,b] Federico Florit,[a] Andrea Mazzei,[a] Stefano Buzzaccaro[a] Barbara Rossi,[c,d] Roberto Piazza,[a] Renato Rota[a] and Luigi De Nardo.[a,b]*

[a] Politecnico di Milano, Department of Chemistry, Materials and Chemical Engineering "G. Natta".

[b] INSTM - Local Unit Politecnico di Milano.

[c] Elettra–Sincrotrone Trieste and INSTM Local Unit, Strada Statale 14 km 163.5, Area Science Park, 34149 Trieste, Italy.

[d] Department of Physics, University of Trento, Via Sommarive 14, 38123 Povo, Trento, Italy

Table of content

S1. UNIFAC model equations and UNIFAC-LLE parameters for the ternary mixture of water, 1,2-dichlorobenzene and ethanol 4

Table S1. Group-contribution parameters for the ternary system DCB-water-ethanol. 7

Table S2. Groups contained in each molecule: aromatic carbon and hydrogen atom (CArH), aromatic carbon and chlorine atom (CArCl), water (H2O), carbon and three hydrogen atoms (CH3), carbon and two hydrogen atoms (CH2), hydroxyl group (OH). 8

S2. Miscibility studies on ternary mixtures of water, 1,2-dichlorobenzene and ethanol. UNIFAC-LLE model validation. **9**

Table S3. Ternary solvents mixtures prepared for the validation of the UNIFAC model and observation concerning their miscibility. N = non miscibile; Y = miscible. 9

Figure S1. ^{1}H-NMR of organic phase of entry 1. 11

Figure S2. ^{1}H-NMR of aqueous phase of entry 1.......... 11

Figure S3. ^{1}H-NMR of organic phase of entry 2.. 12

Figure S4. ^{1}H-NMR of aqueous phase of entry 2.......... 12

Figure S5. ^{1}H-NMR of organic phase of entry 3.. 13

Figure S6. ^{1}H-NMR of aqueous phase of entry 3.......... 13

Figure S7. ^{1}H-NMR of organic phase of entry 4. 14

Figure S8. ^{1}H-NMR of aqueous phase of entry 4.......... 14

Figure S9. ^{1}H-NMR of organic phase of entry 5. 15

Figure S10. ^{1}H-NMR of aqueous phase of entry 5.......... 15

Figure S11. 1H-NMR of pure DMSO-d6 collected in order to evaluate the amount of water in the NMR solvent... 16

Figure S12. 1H-NMR of ethanol 16

Figure S13. Ternary miscibility diagram of H2O, ethanol and 1,2-dichlorobenzene mixtures as computed from UNIFAC-LLE model, expressed as molar fraction. Continuous blue and red lines represent the boundaries of the mixability region (phase separation below these lines). Circles and crosses represent the qualitative empirical validation test. Circles indicate the mixtures which do not lead to phase separation, while crosses indicate mixtures which show phase separation. Squares indicate the mixtures prepared to determine the tie lines (dashed lines) as from 1H-NMR. (colour figure online). The three coloured circles (blue, red and green) correspond to the composition of the three solvent mixtures discussed in Table 1, respectively TM1, TM2 and TM3. 17

Table S4. 1H- Ternary solvent mixtures prepared for the validation of the UNIFAC model by ^{1}H-NMR characterization of each phase 18

S3. Characterization and dispersion in acidic aqueous solution of ZrO_2 nanoparticles. 19

Table S5. Crystalline properties of ZrO_2-NPs determined by XRD and Rietveld refinement.......... 19

Figure S14. HR-TEM images of sample TM2 19

S5. DLS tests of nanoparticle dispersibility in ternary mixtures: how to estimate the cluster sizes and concentration. **20**

Figure S15. Field correlation functions $g_1(\tau)$ of the zirconia dispersions in solvents TM2 (squares). Continuous grey line is an exponential curve with the characteristic decay time $\tau_p = 27.8\ \mu s$. The black dot indicates the value of $g_1(\tau) = w_c$ where the correlation function deviates from the exponential decay expected for monodisperse particles.. 21

Table S6. DLS analysis: cluster sizes and concentration 22

References **23**

S1. UNIFAC model equations and UNIFAC-LLE parameters for the ternary mixture of water, 1,2-dichlorobenzene and ethanol

The mixtures leading to phase separations can give rise to two liquid phases, α and β, characterised by a x_i^α and x_i^β composition, respectively, expressed via the molar fraction of the i-th component. These compositions represent the unknowns of the problem, thus leading to a total of $2NC$ unknowns, where NC is the number of species in the system. In this case 3 species are present, namely DCB, water, and ethanol, thus the total number of unknowns is 6. Once equilibrium is reached at a given temperature, T, pressure, P, and composition $\boldsymbol{x}$ (expressed in vector terms), the same species in the two phases, α and β, must have the same fugacity, namely:

$$\hat{f}_i^\alpha(T,P,\boldsymbol{x}^\alpha) = \hat{f}_i^\beta(T,P,\boldsymbol{x}^\beta)\#(S1)$$

The fugacity of the i-th component in a liquid mixture (phase k) can be expressed as:

$$\hat{f}_i^k(T,P,\boldsymbol{x}^k) = \gamma_i^k(T,P,\boldsymbol{x}^k) \cdot f_i^k(T,P) \cdot x_i^k\#(S2)$$

where γ_i^k is the activity coefficient of the species in the mixture, and f_i^k the fugacity of the pure species in liquid phase. Therefore, at equilibrium, it must be:

$$\gamma_i^\alpha(T,P,\boldsymbol{x}^\alpha) \cdot x_i^\alpha = \gamma_i^\beta(T,P,\boldsymbol{x}^\beta) \cdot x_i^\beta\#(S3)$$

This will enforce a number of equations equal to 3. As a consequence of mass conservation, the stoichiometric relationships must hold true:

$$\sum_{i}^{NC} x_i^{\alpha} = 1 \#(S4)$$

$$\sum_{i}^{NC} x_i^{\beta} = 1 \#(S5)$$

enforcing 2 additional equations. The total number of unknowns is 6, while 5 equations are available, thus there is still 1 degree of freedom for a ternary mixture. It is enough to set (arbitrarily) one of the molar fractions in one of the phases. By parametrically varying this degree of freedom one can obtain all the possible mixtures giving rise to phase separation in a ternary mixture.

To solve the set of Equations (S3), (S4), and (S5), equipped with the proper fixed molar fraction, a model is required to express the functional dependency of the activity coefficients on temperature, pressure, and composition of the phase considered. The UNIFAC-LLE model aims at doing so. Each activity coefficient expression is split into a combinatorial and a residual part:[1]

$$\ln \gamma_i^k(T,P,\boldsymbol{x}^k) = \ln \gamma_i^C(\boldsymbol{x}^k) + \ln \gamma_i^R(T,\boldsymbol{x}^k) \#(S6)$$

The combinatorial part is the part of the activity coefficient described by the pure-species characteristics based on their chemical structure; the value is computed knowing which groups form each molecule in the mixture and the corresponding group-contributions are

combined by means of the molar fraction of each species. The residual part aims at describing the interaction between the various groups by means of binary-interaction parameters (no higher order parameters are necessary);[2] furthermore, the residual part describes the dependency of the activity coefficient on temperature. The pressure dependency is not considered by this model. The equations describing the equilibrium by means of the UNIFAC model are highly nonlinear, thus a numerical solution is needed.

In the following the explicit dependency of the variables on temperature, pressure, and composition will be dropped for clarity purposes. The combinatorial part is expressed as:

$$\ln \gamma_i^C = \ln \frac{\phi_i}{x_i} + \frac{z}{2} q_i \ln \frac{\theta_i}{\phi_i} + l_i - \frac{\phi_i}{x_i} \sum_j^{NC} x_j l_j \#(S7)$$

where the parameters ϕ_i and θ_i are characteristics of the pure species considered and are indications of area and volume fractions, respectively. l_i is an ensemble of pure-species variables z is a characteristic constant equal to 10 for the UNIFAC model. The aforementioned parameters can be expressed as:

$$\phi_i = \frac{r_i x_i}{\sum_j^{NC} r_j x_j} \#(S8)$$

$$\theta_i = \frac{q_i x_i}{\sum_j^{NC} q_j x_j} \#(S9)$$

$$l_i = \frac{z}{2}(r_i - q_i) - (r_i - 1)\#(S10)$$

where r_i and q_i are representative of the molecule van der Waals volumes and surface areas, expressed by means of group contributions as:

$$r_i = \sum_{k}^{NG_i} \nu_{ki} R_k \#(S11)$$

$$q_i = \sum_{k}^{NG_i} \nu_{ki} Q_k \#(S12)$$

where NG_i is the number of different groups in molecule i, ν_{ki} the number of groups of type k in the i-th molecule, and R_k and Q_k represent the group contributions to molecular volume and area, respectively. It should be noted that only pure species parameters characterise the combinatorial part of the activity coefficient.

The residual part of the activity coefficient takes into account binary interactions between the molecules in the mixture. No further (ternary) parameter is necessary to take into account the interaction of three molecules together, even for systems with three or more components.[2] The residual part is expressed as:

$$\ln \gamma_i^R = \sum_{k}^{NG_i} \nu_{ki} \left(\ln \Gamma_k - \ln \Gamma_k^{(i)}\right) \#(S13)$$

where

$$\ln \Gamma_k = Q_k \left[1 - \ln \left(\sum_{m}^{NG_{tot}} \Theta_m \Psi_{mk} \right) - \sum_{m}^{NG_{tot}} \frac{\Theta_m \Psi_{km}}{\sum_{n}^{NG_{tot}} \Theta_n \Psi_{nm}} \right] \#(S14)$$

$$\Theta_m = \frac{Q_m X_m}{\sum_{n}^{NG_{tot}} Q_n X_n} \#(S15)$$

$$\Psi_{mn} = \exp\left(- \frac{a_{mn}}{T} \right) \#(S16)$$

NG_{tot} is the total number of different group types in the mixture, X_m is the molar fraction of the m-th group in the mixture, and a_{mn} is the group-interaction parameter. The quantity $\ln \Gamma_k^{(i)}$ (which can be seen as the interactions of the groups within the same molecule) is computed as from Equation (s8), but considering a fictitious solution made of molecules of species i only. Ultimately, the interaction between different groups is taken into account by a_{mn}, while all other parameters are characteristic of the pure species.

The set of the parameters (a_{mn}, R_k, Q_k) is the one characteristic of UNIFAC-LLE. The equations defined here and in section 2.3.1 was numerically solved using the group-contribution parameters reported in Table S1 for the groups present in the ternary mixture of 1,2-dichlorobenzene, water, and ethanol. The groups constituting each molecule are reported in Table S2. For the sake of example, DCB is formed by 4 aromatic carbons with and hydrogen atom (CArH) and 4 aromatic carbons with a chlorine atom (CArCl).

Table S1. Group-contribution parameters for the ternary system DCB-water-ethanol.[3]

a_{mn}							R_m	Q_m
n / m	ACH	ACCl	H_2O	CH_3	CH_2	OH		
ACH	0	777.8	390.7	-59.06	-59.06	1989	1.1562	0.8440
ACCl	-878.1	0	859.4	156.5	156.5	703.9	0.5313	0.4000
H_2O	-97.27	372.8	0	342.4	342.4	-122.4	0.9200	1.400
CH_3	924.8	-114.8	1300	0	0.000	644.6	0.9011	0.8480
CH_2	924.8	-114.8	1300	0.000	0	644.6	0.6744	0.5400
OH	-597.1	-9.210	28.73	328.2	328.2	0	1.000	1.200

Table S2. Groups contained in each molecule: aromatic carbon and hydrogen atom ($C_{Ar}H$), aromatic carbon and chlorine atom ($C_{Ar}Cl$), water (H_2O), carbon and three hydrogen atoms (CH_3), carbon and two hydrogen atoms (CH_2), hydroxyl group (OH).

	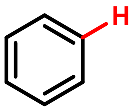 $C_{Ar}H$	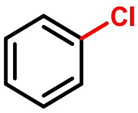 $C_{Ar}Cl$	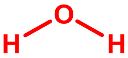 H_2O	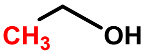 CH_3	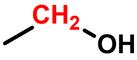 CH_2	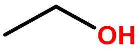 OH
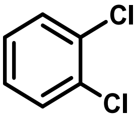 DCB	4	2	0	0	0	0
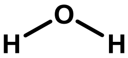 Water	0	0	1	0	0	0
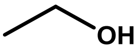 EtOH	0	0	0	1	1	1

S2. Miscibility studies on ternary mixtures of water, 1,2-dichlorobenzene and ethanol. UNIFAC-LLE model validation.

Table S3. Ternary solvents mixtures prepared for the validation of the UNIFAC model and observation concerning their miscibility. N = non miscibile; Y = miscible.

ω_{DCB} (%)	ω_{H2O} (%)	ω_{EtOH} (%)	Predicted miscibility	Observed miscibility
19.00	24.00	57.00	N	N
37.00	15.00	48.00	N	N
60.00	10.00	30.00	N	N
82.00	4.00	14.00	N	N
20.00	30.00	50.00	N	N
90.00	1.00	9.00	N	Y
50.00	12.00	38.00	N	N
70.00	9.00	21.00	N	N

88.00	3.00	9.00	N	N
46.01	10.80	43.20	N	N
47.33	10.53	42.13	N	N
10.00	40.00	50.00	N	N
9.00	45.00	46.00	N	N
12.00	36.00	52.00	N	N
25.57	20.40	54.03	N	N
82.75	1.51	15.74	Y	Y
15.00	21.00	64.00	Y	Y
35.00	10.00	55.00	Y	Y
60.00	2.00	38.00	Y	Y
82.00	1.00	17.00	Y	Y
50.00	4.00	46.00	Y	Y
70.00	1.00	29.00	Y	Y
86.00	1.00	13.00	Y	Y
45.91	5.68	48.42	Y	Y

44.95	7.66	47.40	Y	Y
46.43	7.45	46.12	Y	Y
45.50	9.30	45.20	Y	N
46.90	9.06	44.04	Y	N
4.00	40.00	56.00	Y	Y
2.00	41.00	57.00	Y	Y
10.00	30.00	60.00	Y	Y
65.00	3.00	32.00	Y	Y
24.82	18.92	56.27	Y	N
26.07	14.92	59.01	Y	Y
10.52	19.96	69.52	Y	Y
22.79	10.19	67.02	Y	Y
12.84	9.77	77.39	Y	Y
5.76	4.72	89.52	Y	Y

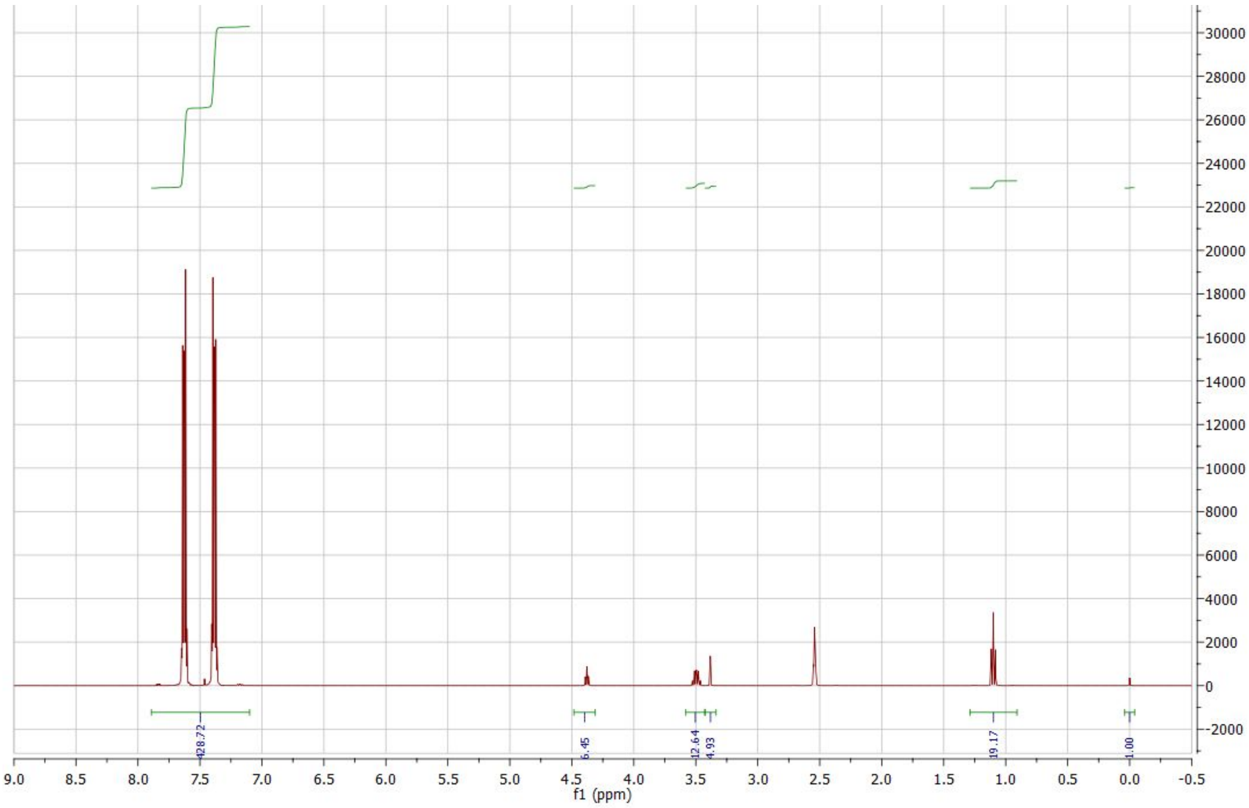

Figure S1. ^{1}H-NMR of organic phase of entry 1.

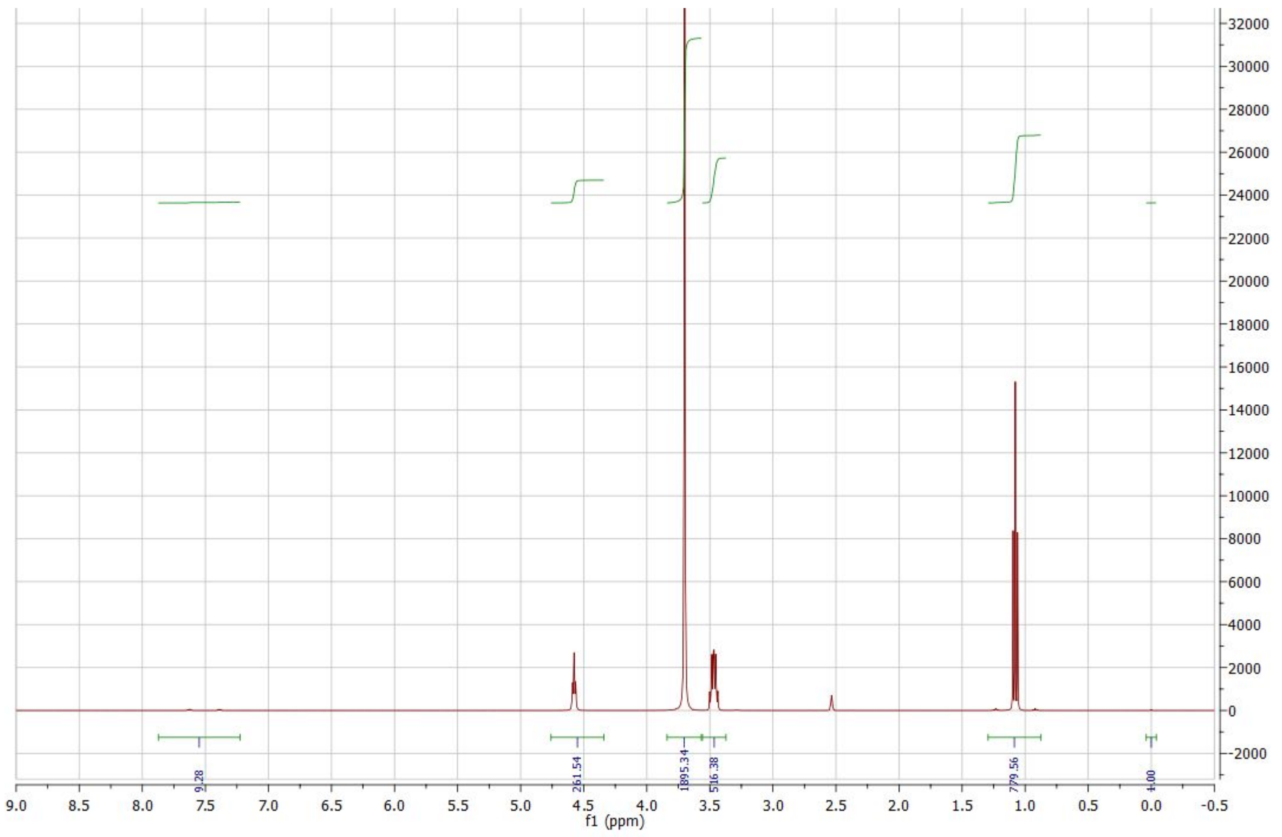

Figure S2. ^{1}H-NMR of aqueous phase of entry 1.

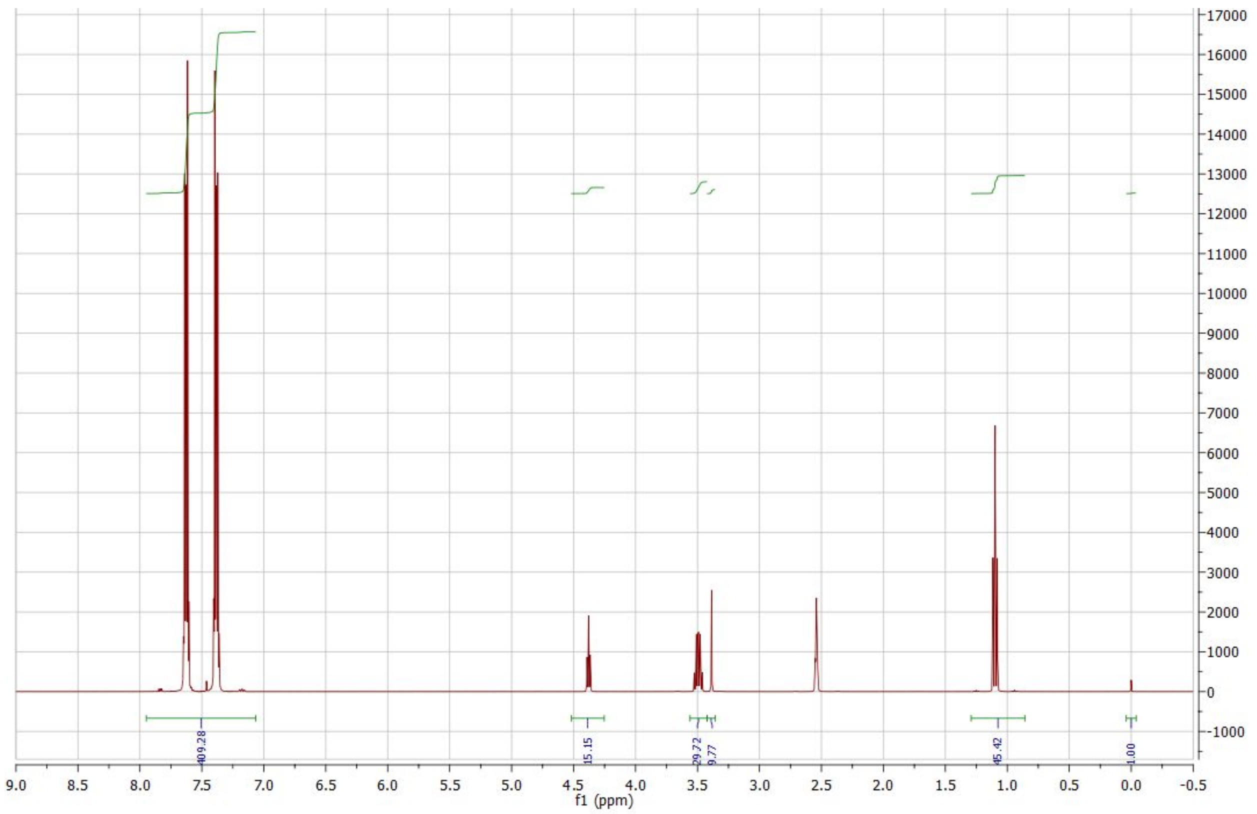

Figure S3. ^{1}H-NMR of organic phase of entry 2.

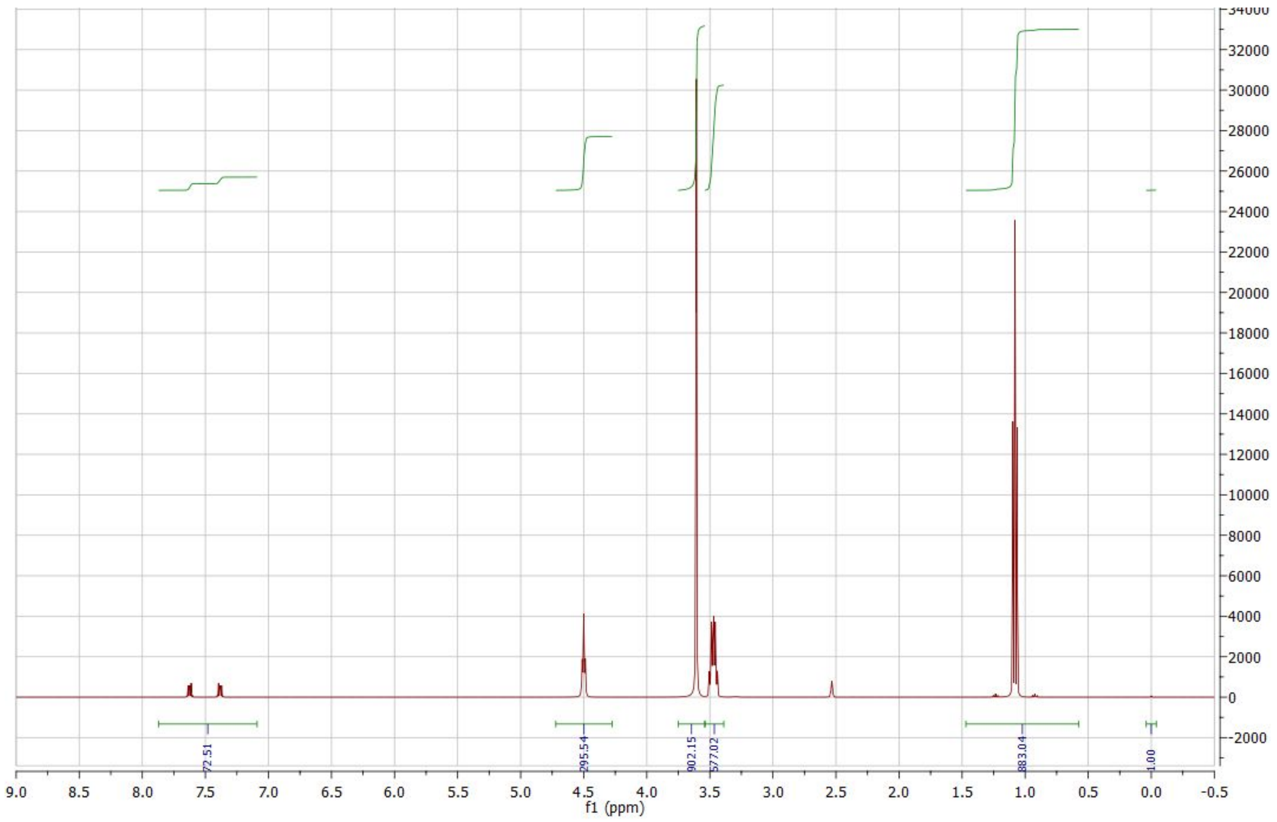

Figure S4. ^{1}H-NMR of aqueous phase of entry 2.

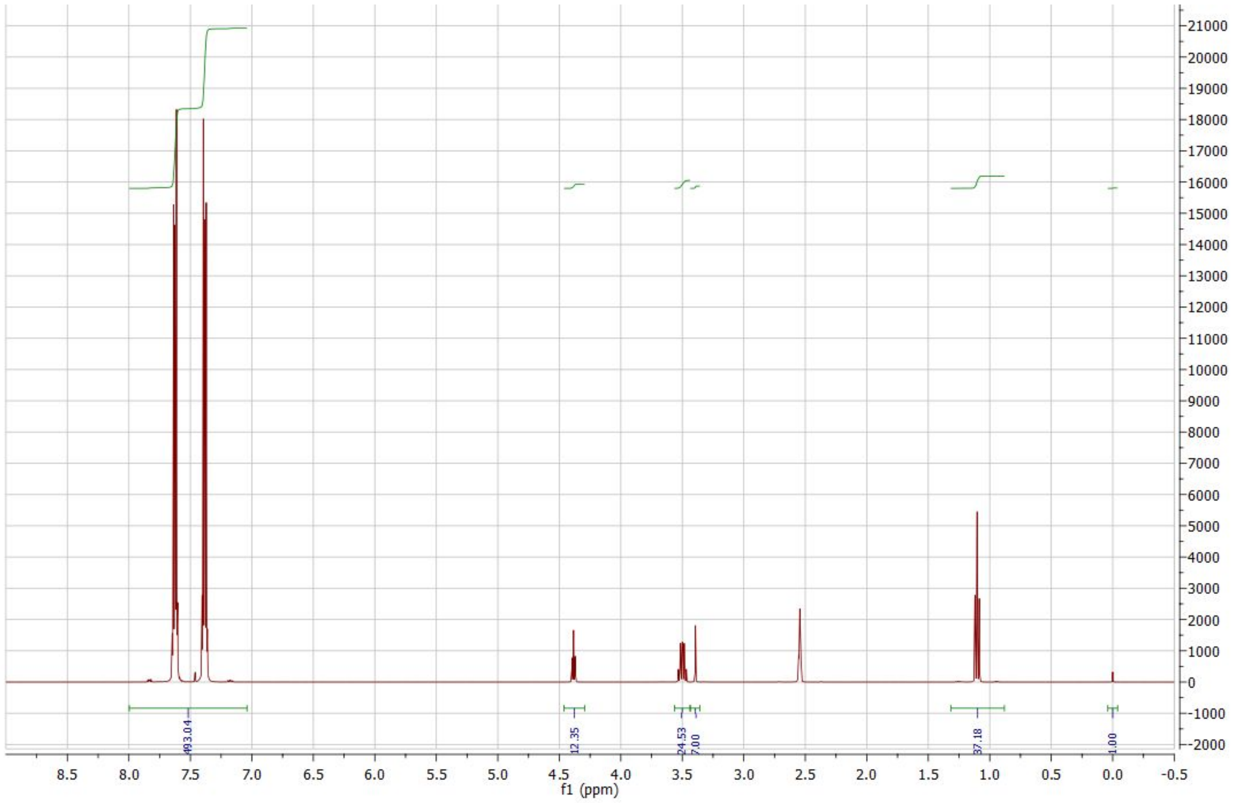

Figure S5. ^{1}H-NMR of organic phase of entry 3.

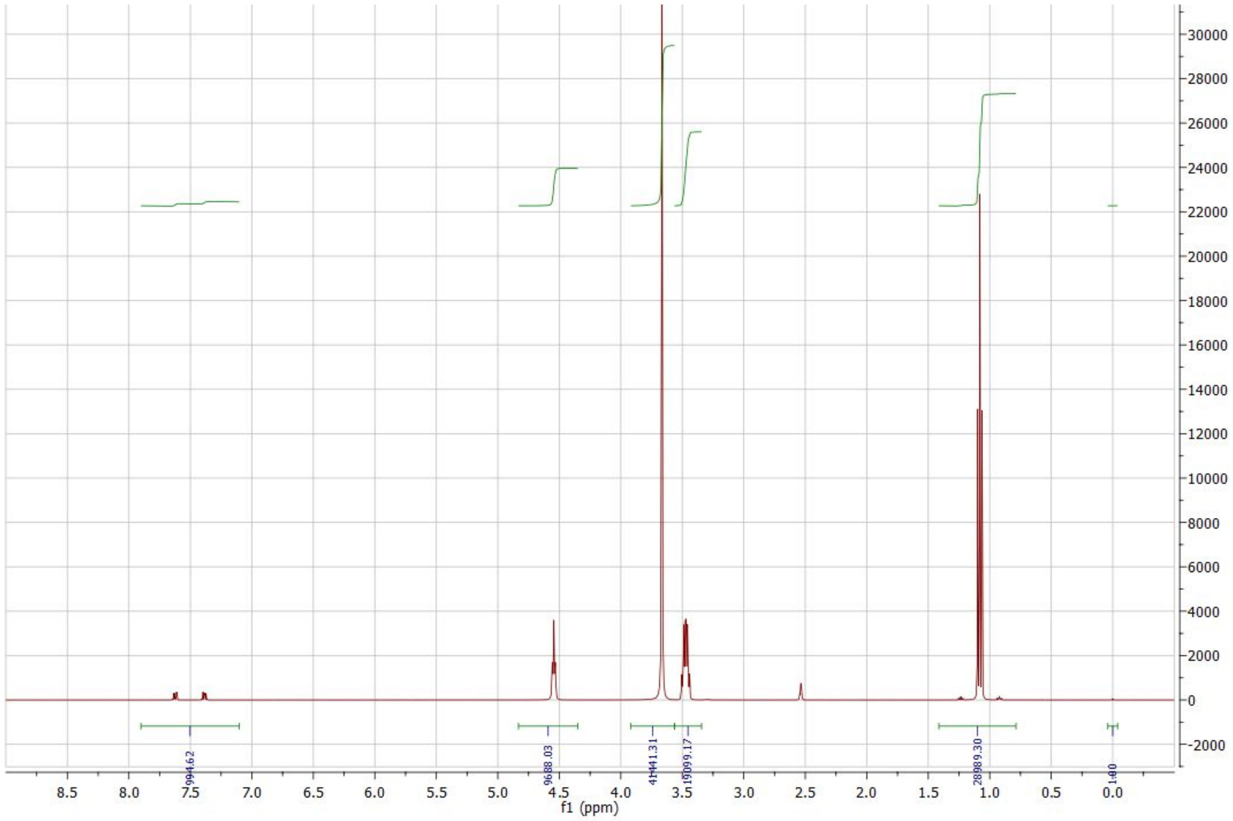

Figure S6. ^{1}H-NMR of aqueous phase of entry 3.

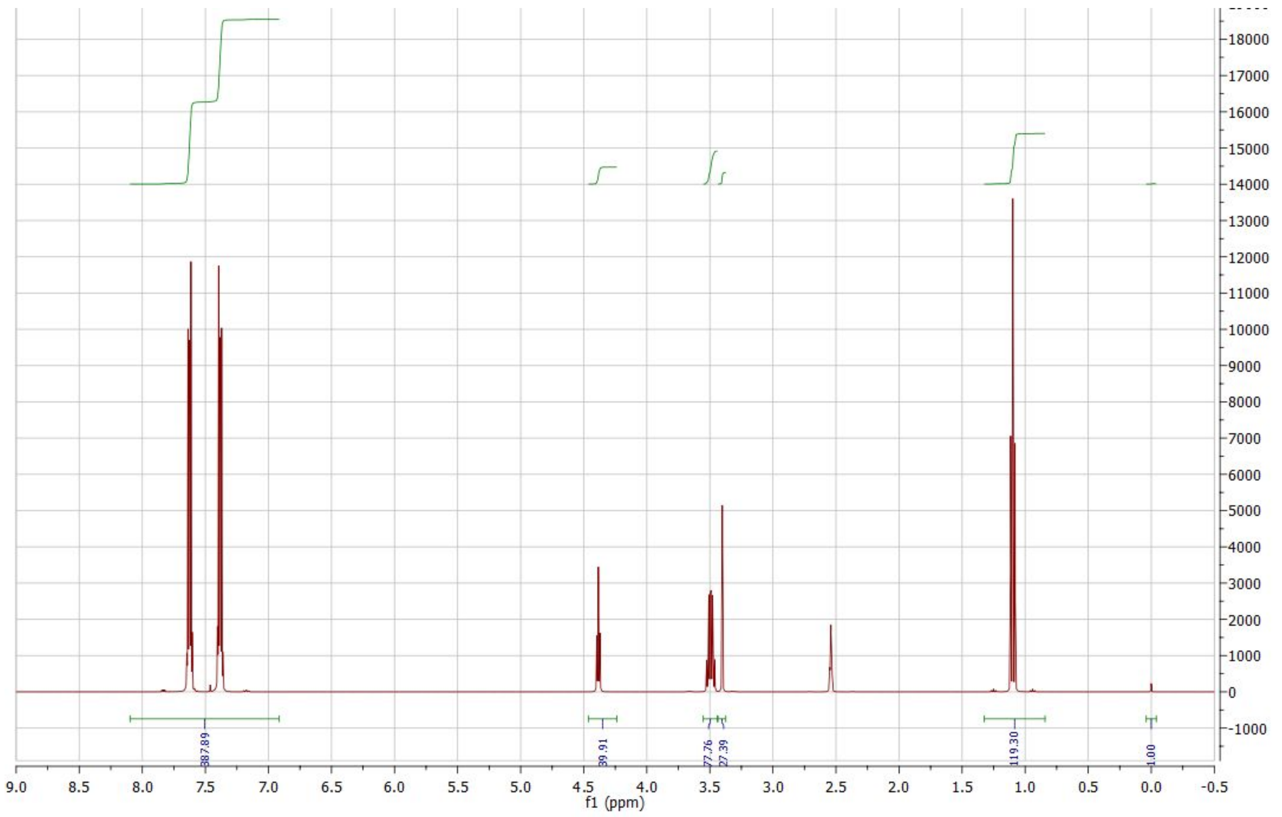

Figure S7. ^{1}H-NMR of organic phase of entry 4.

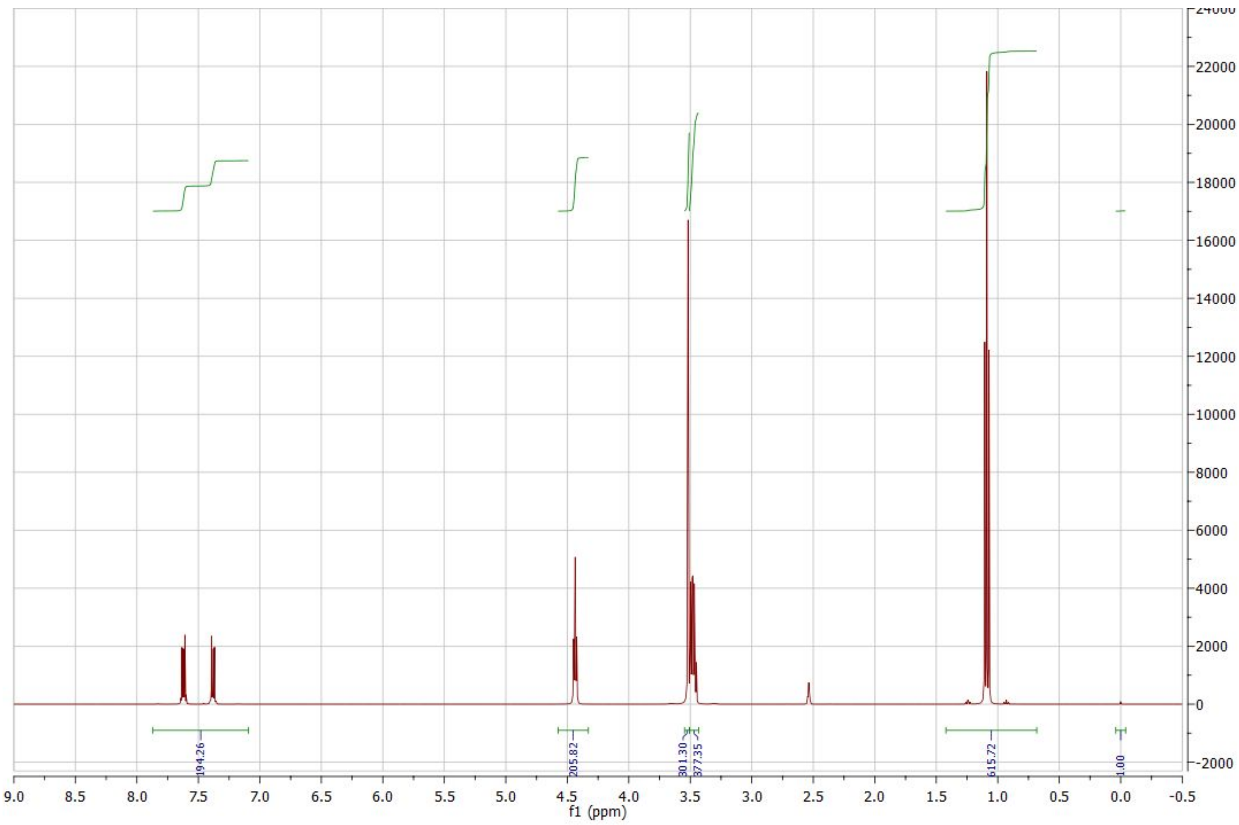

Figure S8. ^{1}H-NMR of aqueous phase of entry 4.

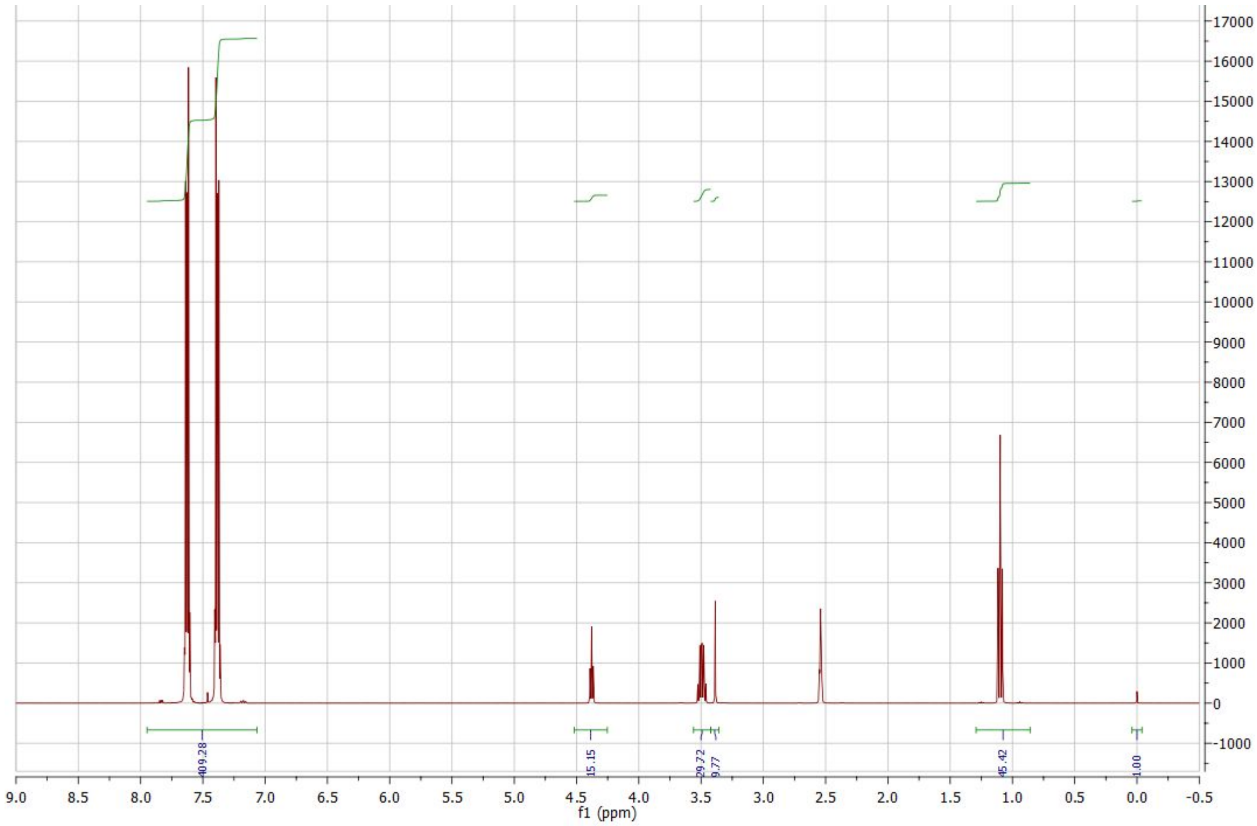

Figure S9. ^{1}H-NMR of organic phase of entry 5.

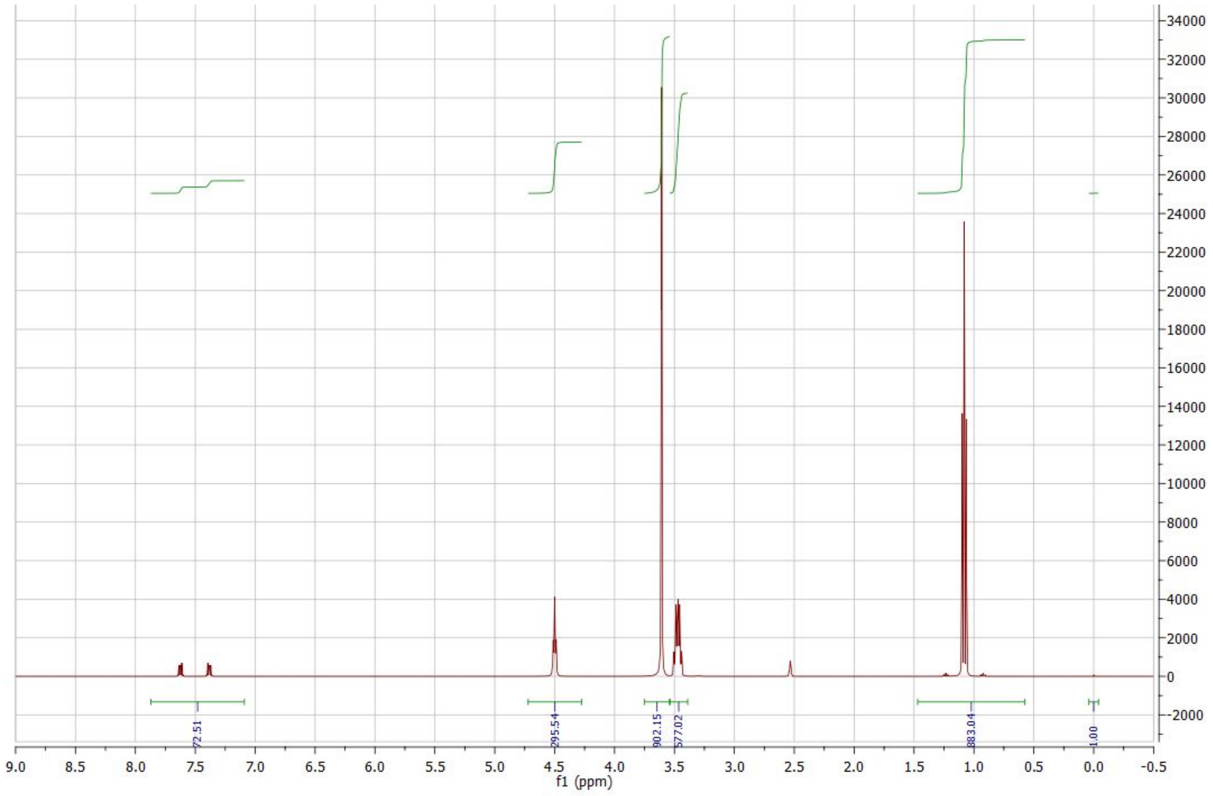

Figure S10. ^{1}H-NMR of aqueous phase of entry 5.

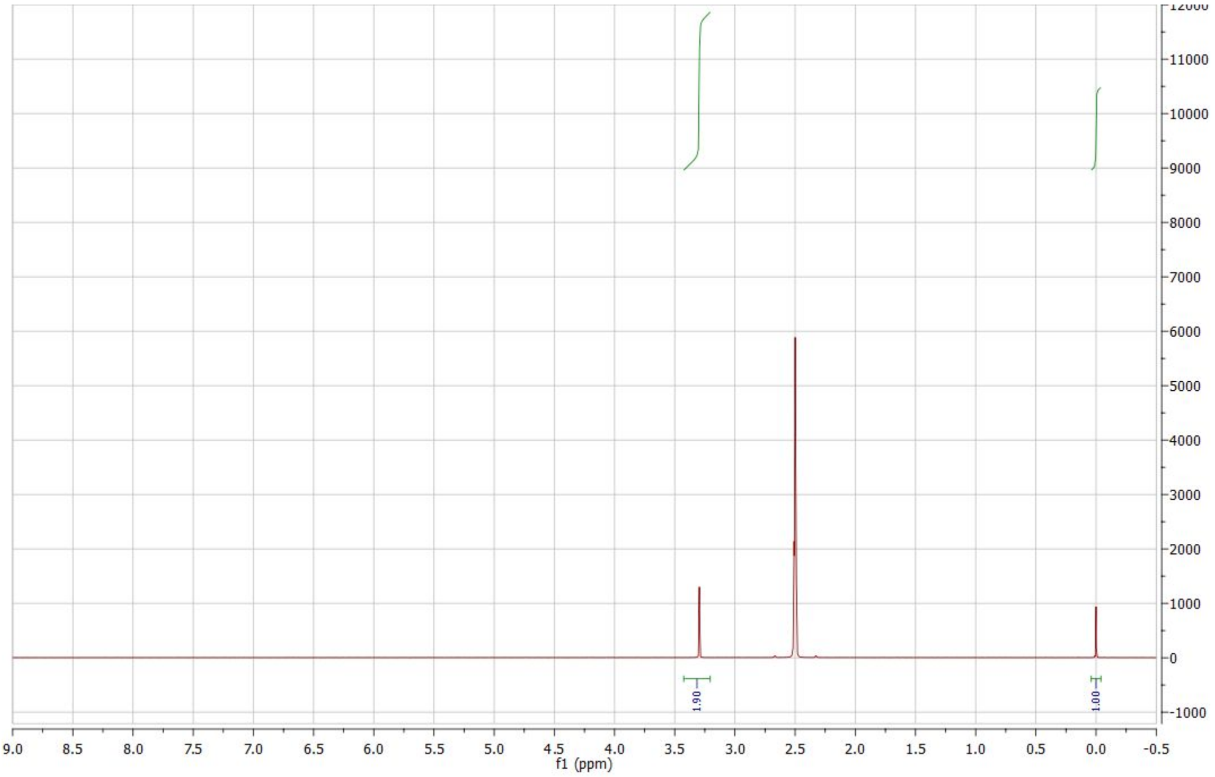

Figure S11. 1H-NMR of pure DMSO-d6 collected in order to evaluate the amount of water in the NMR solvent.

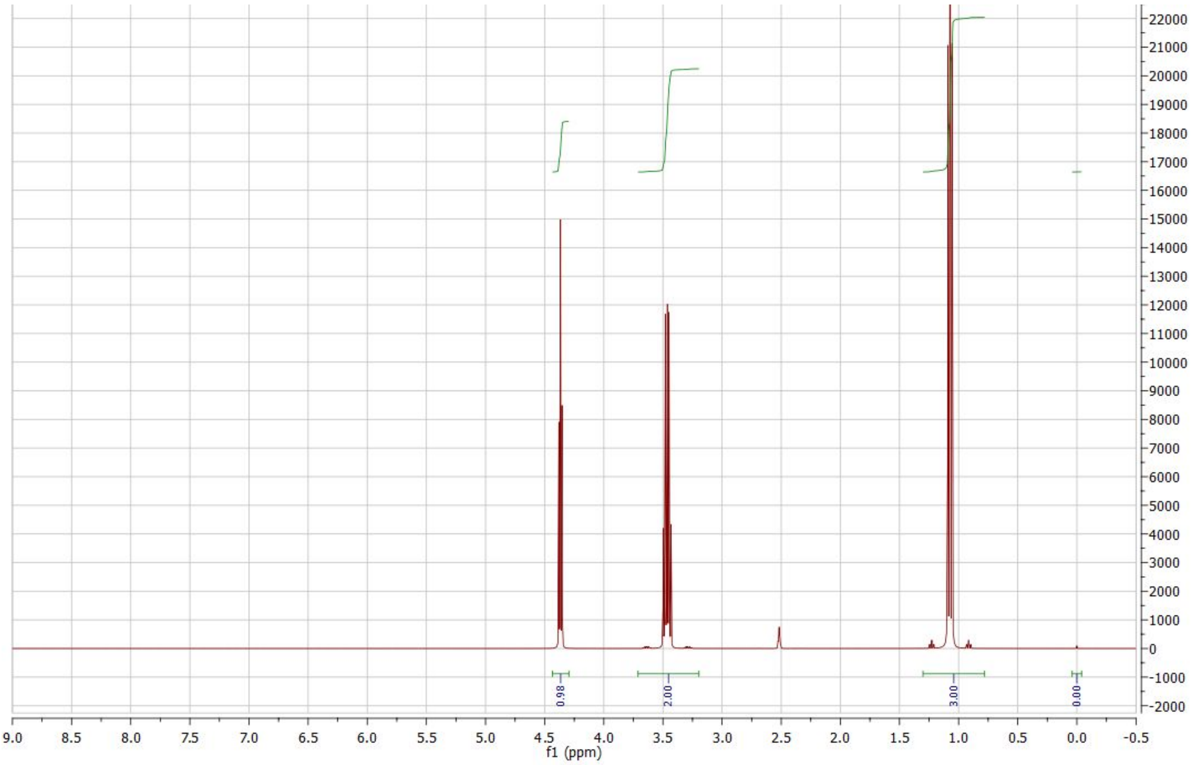

Figure S12. ^{1}H-NMR of ethanol

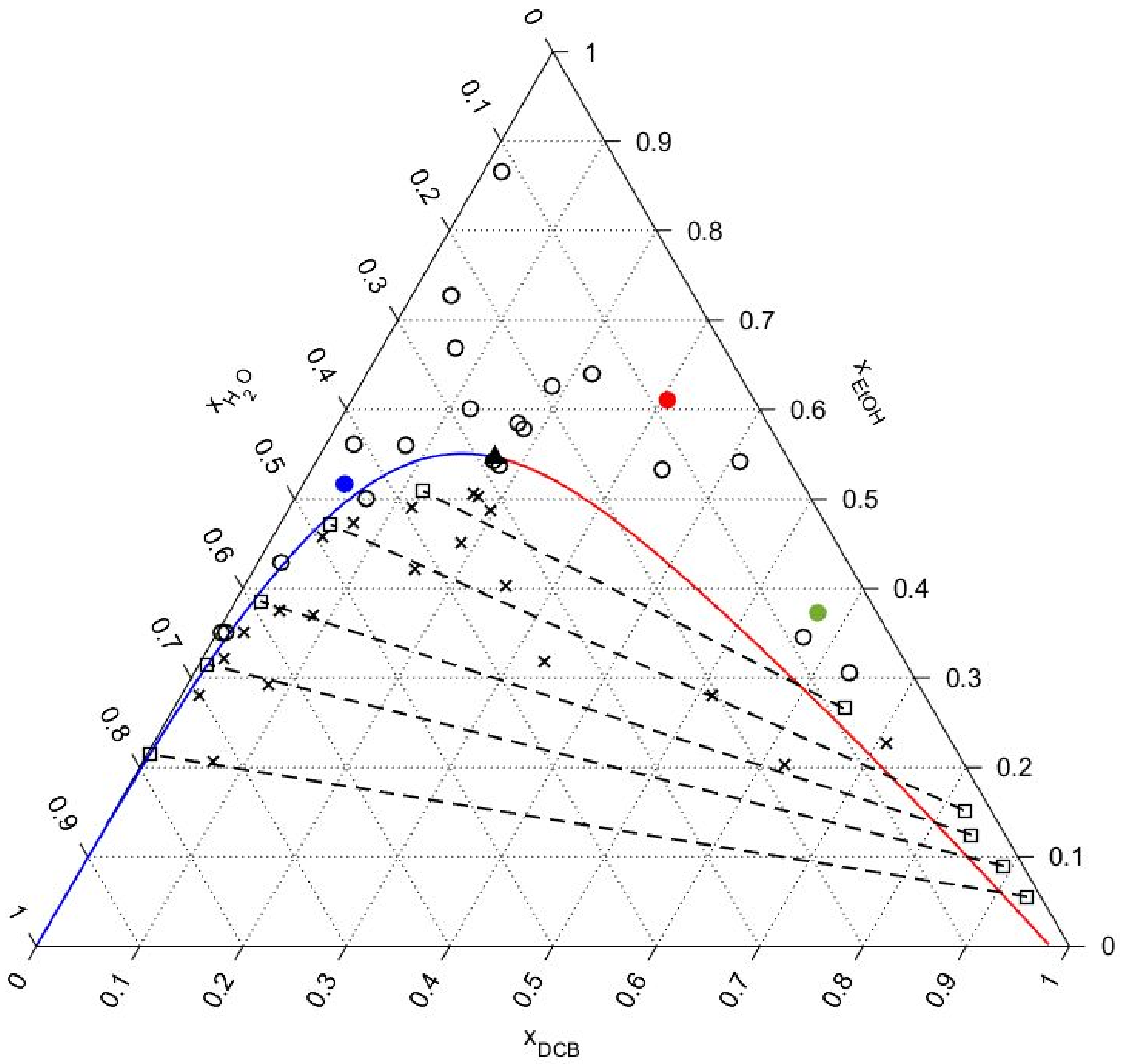

Figure S13. Ternary miscibility diagram of H2O, ethanol and 1,2-dichlorobenzene mixtures as computed from UNIFAC-LLE model, expressed as molar fraction. Continuous blue and red lines represent the boundaries of the mixability region (phase separation below these lines). Circles and crosses represent the qualitative empirical validation test. Circles indicate the mixtures which do not lead to phase separation, while crosses indicate mixtures which show phase separation. Squares indicate the mixtures prepared to determine the tie lines (dashed lines) as from ^{1}H-NMR. (colour figure online). The three coloured circles (blue, red

and green) correspond to the composition of the three solvent mixtures discussed in Table 1, respectively TM1, TM2 and TM3.

Table S4. Ternary solvent mixtures prepared for the validation of the UNIFAC model by ^{1}H-NMR characterization of each phase.

Entry	Initial			Measured ω of the obtained phases			Predicted ω of the obtained phases		
	ωDCB (%)	ωH_2O (%)	ωEtOH (%)	ωDCB (%)	ωH_2O (%)	ωEtOH (%)	ωDCB (%)	ωH_2O (%)	ωEtOH (%)
1	30.53	40.17	29.30	98.00	0.17	1.83	87.78	1.64	10.57
				1.16	58.07	40.77	1.35	59.88	38.77
2	31.36	25.13	43.52	95.14	0.45	4.41	79.52	2.76	17.73
				10.95	33.32	55.73	7.34	36.23	56.43
3	31.54	31.23	37.23	96.71	0.25	3.05	83.76	2.17	14.07
				4.28	43.65	52.07	3.60	46.71	49.69
4	45.52	45.73	15.26	94.18	0.36	5.46	75.51	3.33	21.16
				19.19	22.98	57.83	11.61	29.17	59.22
5	50.56	10.00	39.44	87.37	1.41	11.23	66.97	4.65	28.38
				37.01	13.98	49.01	21.51	19.65	58.84

S3. Characterization and dispersion in acidic aqueous solution of ZrO_2 nanoparticles.

Table S5. Crystalline properties of ZrO_2-NPs determined by XRD and Rietveld refinement.

	m-ZrO_2 fraction (wt %)	Crystallite size		χ^2	GOF
		m-ZrO_2 (nm)	t-ZrO_2 (nm)		
ZrO_2-NPs	69	3.7	3.9	1.76	1.33

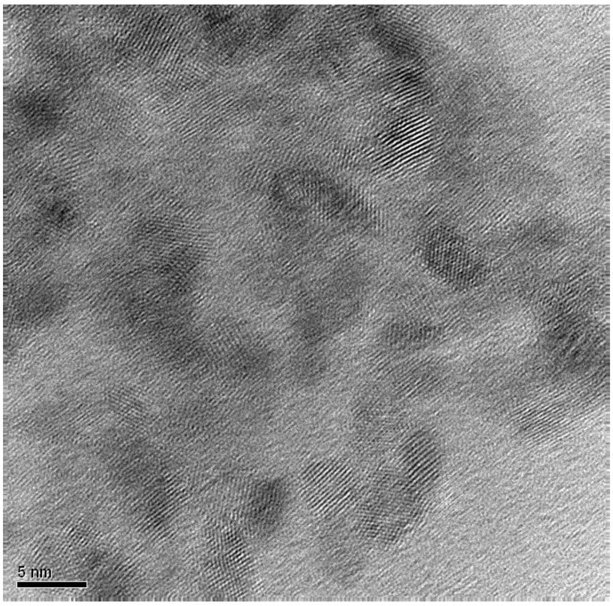

Figure S14. HR-TEM images of sample TM2.

S5. DLS tests of nanoparticle dispersibility in ternary mixtures: how to estimate the cluster sizes and concentration.

In the case of polydisperse samples, the field autocorrelation function $g_1(\tau)$ is a sum of the exponential decays corresponding to each of the species in the population:

$$g_1(\tau) = \sum_{i=1}^{n} w_i \exp\left(-\tau/\tau_i\right), \qquad (S17)$$

where the weights w_i are proportional to the relative scattering from each species and the characteristic decay times τ_i are proportional to the species size R_i.

Exploiting the properties of the exponential, it is easy to demonstrate that the integral of the correlation function:

$$\int_0^\infty d\tau g_1(\tau) = \sum_{i=1}^{n} w_i \tau_i = \tau_{AVG} \#(S18)$$

is equal to the intensity-averaged decay time τ_{AVG}.

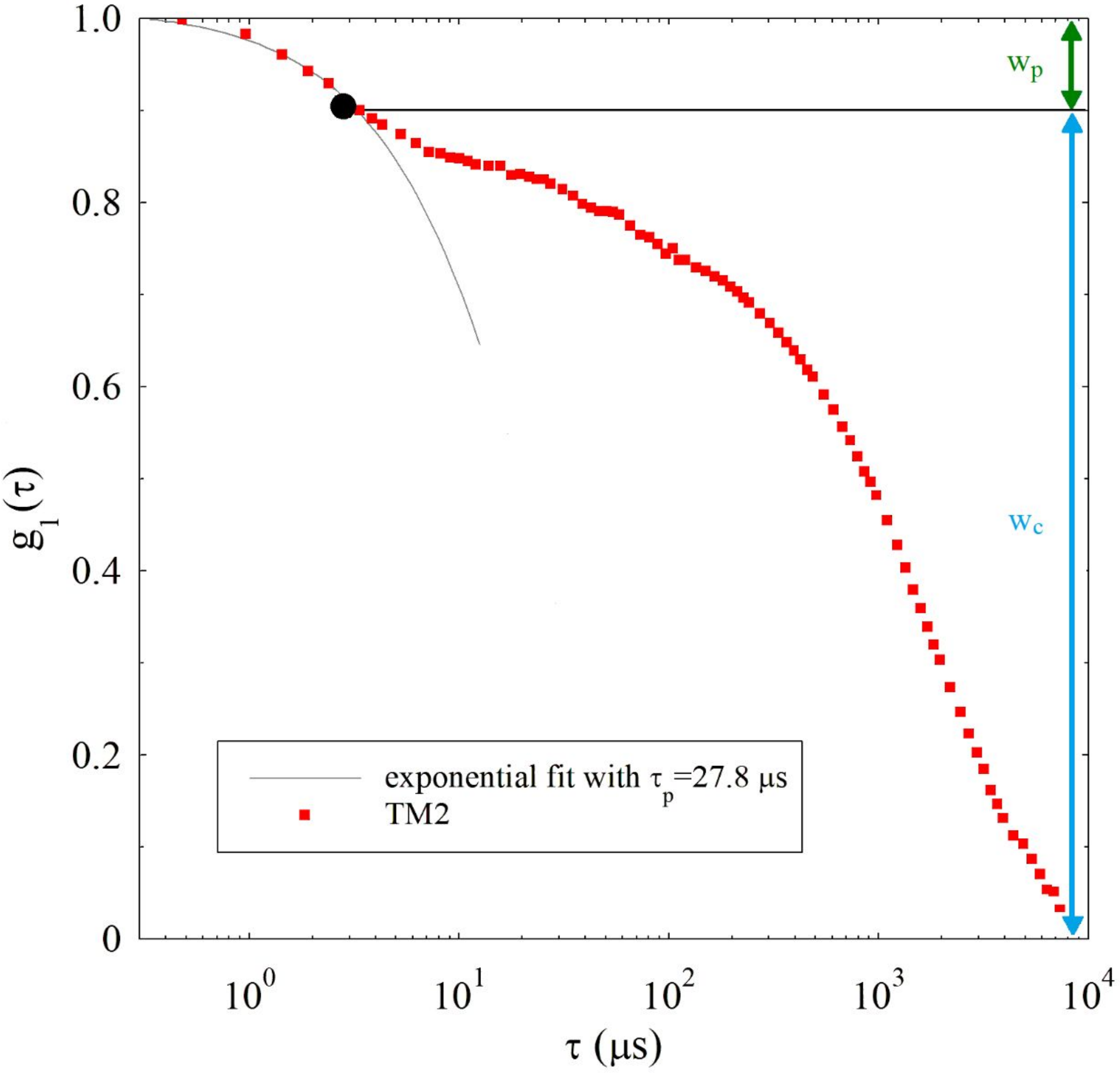

Figure S15. Field correlation functions $g_1(\tau)$ of the zirconia dispersions in solvents TM2 (squares). Continuous grey line is an exponential curve with the characteristic decay time $\tau_p = 27.8\ \mu s$. The black dot indicates the value of $g_1(\tau) = w_c$ where the correlation function deviates from the exponential decay expected for monodisperse particles.

To estimate the typical cluster size R_c and concentration c_c we make the simplifying assumption that in our samples are present only two populations, the particles and the clusters.

In this case Eq.S18 will become:

$$\int_0^{\infty} d\tau g_1(\tau) = w_p \tau_p + w_c \tau_c = \tau_{AVG} \#(S19)$$

Where the subscripts p and c refer to the particles and clusters respectively.

The τ_p can be easily obtained by fitting the first part of the field correlation function for the nanoparticles in the original aqueous solvent (H_2O + 0.1 M NaCl). Our fit gives a value of $\tau_p = 27.8\ \mu s$, which corresponds to a typical particle radius $R_p = 4.66\ nm$.

The weights w_p and w_c can be estimated by looking when the correlation function departs significatively from the single exponential behaviour expected for monodisperse particles (see figure S15). Once τ_{AVG}, τ_p, w_p and w_c are known, the value of τ_c can be easily calculated by inverting Eq.2. To estimate the fraction of particles associated in clusters is useful to recall that:

$$w_i \propto c_i(R_i)^3 \# (S20)$$

The fraction of particles associated in cluster is then:

$$\frac{c_c}{c_p} = \frac{w_c}{w_p}\left(\frac{R_p}{R_c}\right)^3 \# (S21)$$

The results are reported in the Table S6.

Table S6. DLS analysis: cluster sizes and concentration

Solvent	w_p	w_c	τ_{AVG} (μs)	τ_c (μs)	R_c (nm)	c_c/c_p
**Water+0.1M HCl**	0.8	0.2	162.811	703	118	$1.5*10^{-5}$
**TM1**	0.2	0.8	1507	1877	314	$1.3*10^{-5}$
**TM2**	0.1	0.9	1664	1846	309	$3*10^{-5}$

References

(1) Fredenslund, A.; Jones, R. L.; Prausnitz, J. M. Group-Contribution Estimation of Activity Coefficients in Nonideal Liquid Mixtures. *AIChE J.* **1975**, *21* (6), 1086–1099. https://doi.org/10.1002/aic.690210607.

(2) Abrams, D. S.; Prausnitz, J. M. Statistical Thermodynamics of Liquid Mixtures: A New Expression for the Excess Gibbs Energy of Partly or Completely Miscible Systems. *AIChE J.* **1975**, *21* (1), 116–128. https://doi.org/10.1002/aic.690210115.

(3) Magnussen, T.; Rasmussen, P.; Fredenslund, A. Unifac Parameter Table for Prediction of Liquid-Liquid Equilibria. *Ind. Eng. Chem. Process Des. Dev.* **1981**, *20* (2), 331–339. https://doi.org/10.1021/i200013a024.
